# Harnessing the potential of CAR-T cell therapy: progress, challenges, and future directions in hematological and solid tumor treatments

**DOI:** 10.1186/s12967-023-04292-3

**Published:** 2023-07-07

**Authors:** Gunjan Dagar, Ashna Gupta, Tariq Masoodi, Sabah Nisar, Maysaloun Merhi, Sheema Hashem, Ravi Chauhan, Manisha Dagar, Sameer Mirza, Puneet Bagga, Rakesh Kumar, Ammira S. Al-Shabeeb Akil, Muzafar A. Macha, Mohammad Haris, Shahab Uddin, Mayank Singh, Ajaz A. Bhat

**Affiliations:** 1 Department of Medical Oncology (Lab.), Dr. BRAIRCH, All India Institute of Medical Sciences (AIIMS), New Delhi, Delhi 110029 India; 2 Laboratory of Cancer Immunology and Genetics, Sidra Medicine, Doha, Qatar; 3 Department of Diagnostic Imaging, St. Jude Children’s Research Hospital, Memphis, TN USA; 4 National Center for Cancer Care and Research, Hamad Medical Corporation, 3050 Doha, Qatar; 5 Department of Human Genetics, Sidra Medicine, Doha, Qatar; 6 Shiley Eye Institute, University of California San Diego, San Diego, CA USA; 7 Department of Chemistry, College of Sciences, United Arab Emirates University, Al-Ain, United Arab Emirates; 8 School of Biotechnology, Shri Mata Vaishno Devi University, Katra, Jammu and Kashmir 182320 India; 9 Department of Human Genetics-Precision Medicine in Diabetes, Obesity and Cancer Program, Sidra Medicine, P.O. Box 26999, Doha, Qatar; 10 Watson-Crick Centre for Molecular Medicine, Islamic University of Science and Technology, Pulwama, Jammu and Kashmir India; 11 Center for Advanced Metabolic Imaging in Precision Medicine, Department of Radiology, Perelman School of Medicine, University of Pennsylvania, Philadelphia, USA; 12 Laboratory Animal Research Center, Qatar University, Doha, Qatar; 13 Translational Research Institute, Academic Health System, Hamad Medical Corporation, P.O. Box 3050, Doha, Qatar

**Keywords:** CAR-T cell therapy, Immunotherapy, Tumor antigens, Antigen escape, Cytokine release syndrome, Hematological malignancy, Solid tumor

## Abstract

Traditional cancer treatments use nonspecific drugs and monoclonal antibodies to target tumor cells. Chimeric antigen receptor (CAR)-T cell therapy, however, leverages the immune system's T-cells to recognize and attack tumor cells. T-cells are isolated from patients and modified to target tumor-associated antigens. CAR-T therapy has achieved FDA approval for treating blood cancers like B-cell acute lymphoblastic leukemia, large B-cell lymphoma, and multiple myeloma by targeting CD-19 and B-cell maturation antigens. Bi-specific chimeric antigen receptors may contribute to mitigating tumor antigen escape, but their efficacy could be limited in cases where certain tumor cells do not express the targeted antigens. Despite success in blood cancers, CAR-T technology faces challenges in solid tumors, including lack of reliable tumor-associated antigens, hypoxic cores, immunosuppressive tumor environments, enhanced reactive oxygen species, and decreased T-cell infiltration. To overcome these challenges, current research aims to identify reliable tumor-associated antigens and develop cost-effective, tumor microenvironment-specific CAR-T cells. This review covers the evolution of CAR-T therapy against various tumors, including hematological and solid tumors, highlights challenges faced by CAR-T cell therapy, and suggests strategies to overcome these obstacles, such as utilizing single-cell RNA sequencing and artificial intelligence to optimize clinical-grade CAR-T cells.

## Introduction

Immunotherapy boosts the immune system's ability to fight cancer cells by modulating the capacity of immune cells [1–3]. In the last decade, there have been giant strides in the use of immunotherapy to treat cancer, as evidenced by the approval of both monoclonal antibodies to target different immune system components and adaptive T-cell-based therapies [4]. Surgery, radiation therapy, and chemotherapy are typically recognized as the traditional forms of cancer treatment. However, with its recent clinical successes, immunotherapy has been dubbed the fourth pillar of cancer treatment [5]. Because innate and adaptive immunity consists of a wide variety of cells with various properties able to fight cancer, the essential question was how immunotherapy could be harnessed to develop an effective treatment against cancer [6]. Numerous cancer immunotherapy strategies are currently under investigation, which encompasses immune checkpoint inhibitors, cancer vaccines, immunomodulators, cytokines, monoclonal antibodies, and oncolytic viruses (OVs) [7]. Although many of these approaches have received clinical approval, they each possess inherent limitations that hinder their full therapeutic potential. Consequently, this emphasizes the necessity for pioneering treatments, such as chimeric antigen receptor (CAR)-T cell therapy, to address these constraints. Immune checkpoint inhibitors have emerged as potent allies in cancer treatment, showing remarkable success in several malignancies. However, they have significant limitations, including the development of resistance, immune-related adverse events, and a low response rate in many tumor types. For instance, even in melanoma, where immune checkpoint inhibitors have had the most success, only a subset of patients show a durable response. Cancer vaccines have shown promise in the preclinical setting but have struggled to reproduce those results in the clinic. Often, the immune response they generate is insufficient to overcome the immunosuppressive tumor microenvironment, and they have so far been successful only in a limited number of cancers such as prostate cancer. Immunomodulators, while potent in augmenting immune response, can induce systemic side effects due to their non-specific nature. Additionally, resistance to these agents can develop over time, and they often have a relatively narrow therapeutic window. Monoclonal antibodies have also demonstrated remarkable efficacy in certain cancers. Nevertheless, issues such as off-target toxicity, immunogenicity, resistance, and a lack of response in a significant subset of patients persist. These issues are indicative of the complex nature of cancer and the intricate interplay between the tumor and the immune system, highlighting the need for innovative, targeted therapies such as CAR-T cells. Although CAR-T cell therapy has its limitations, such as cytokine release syndrome and the potential for on-target off-tumor effects, it represents an exciting and promising approach in the field of cancer immunotherapy. Unlike other therapies, CAR-T cells are engineered to specifically recognize and target cancer cells, offering a high degree of specificity [8]. This is achieved by genetically modifying a patient's T cells to express a CAR, which is designed to recognize a specific antigen present on the surface of tumor cells [9]. This unique attribute makes CAR-T cell therapy stand out from other therapies, such as immune checkpoint inhibitors and cancer vaccines, which typically rely on modulating the patient's immune system to fight cancer and often struggle with issues of specificity and efficacy. Furthermore, CAR-T cell therapies have demonstrated unprecedented response rates, particularly in certain hematological malignancies. For example, they have shown impressive results in treating B-cell malignancies such as refractory acute lymphoblastic leukemia (ALL), where other treatment modalities have failed [10]. Moreover, the 'living drug' nature of CAR-T cells, which allows for their expansion and persistence in the patient, offers a sustained antitumor response, a feature not shared by many other therapies, such as monoclonal antibodies. Despite these advantages, it is essential to acknowledge that CAR-T cell therapy is not without its challenges, including the risk of cytokine release syndrome, neurotoxicity, and the potential for 'on-target, off-tumor' effects. However, advancements are continually being made in CAR design and T-cell engineering to improve safety and efficacy.

In summary, CAR T cell therapy's distinctive ability to harness the specificity of adaptive immunity, combined with its potential to provide durable responses in hard-to-treat cancers, presents it as a significant addition to the cancer immunotherapy arsenal.

## Immunotherapy targeting cancer and immune cells

The discovery of the role of immune checkpoint molecules in cancer was a critical moment in the rise of immunotherapy [7]. Immune checkpoint molecules such as PD-1 and CTLA-4, upon activation, bind to their ligands to inhibit excessive expansion of activated T cells. However, tumors overexpress these checkpoint markers to avoid immune surveillance within the tumor microenvironment [11]. In 1996, James Allison and his team discovered that administering antibodies blocking CTLA-4 interaction with CD28 led to increased T-cell activation and tumor rejection [12]. Allison’s research group demonstrated that exhausted T cells upregulated CTLA-4 expression, but the cells were only reversibly exhausted. The discovery that the blockade of supplementary signaling through CTLA-4 could potentially reactivate 'exhausted' cells, ignited an urgency to identify further markers indicative of T-cell exhaustion [12]. This pursuit swiftly led to the uncovering of the PD-1/PD-L1 axis. Since then, the FDA has given approval for clinical use to monoclonal antibodies that inhibit either receptor [13]. Other investigated immune checkpoint molecules include TIM3, LAG3, VISTA, and B7-H3 [14–17]. Antagonistic antibodies against a combination of these markers are also of particular interest, as several combination approaches have demonstrated effective synergies [18, 19]. Immune checkpoint inhibitors have marked a significant shift in the paradigm of cancer treatment, demonstrating considerable success in multiple malignancies. In terms of clinical outcomes, they have shown durable responses and improved survival rates in several types of cancer, including melanoma, lung cancer, and kidney cancer [20]. For example, pembrolizumab and nivolumab, both PD-1 inhibitors, have been particularly effective in treating metastatic melanoma, drastically improving the 5-year survival rate for these patients [21]. However, immune checkpoint inhibitors also have certain limitations that need to be addressed. Firstly, not all patients respond to these treatments. The response rates can vary significantly depending on the cancer type, ranging from about 15–20% in some cancers to over 50% in others. Secondly, some patients may initially respond but then develop resistance over time, leading to disease progression [22]. Furthermore, immune checkpoint inhibitors can cause immune-related adverse events (irAEs), resulting from the overactivation of the immune system. These irAEs can affect any organ system and can sometimes be severe or life-threatening [23]. Immune checkpoint inhibitors work by blocking inhibitory pathways of the immune system, thereby enhancing the ability of immune cells to function more effectively. While they do not directly facilitate the immune system's selective identification and elimination of cancer cells, they play a crucial role in amplifying the immune response against cancer [13].

OVs are another class of immune therapy that uses self-replicating viruses to kill cancer cells via inflammation and cellular death due to exposure to cancer-associated antigens. OVs have been engineered to show improved tumor tropism [24]. These viruses are modified in vitro to infect tumors; once they infect a tumor, they create a proinflammatory environment and release the tumor antigen through the lytic pathway [24]. Additionally, viruses can be engineered to express antigens in tumor cells upon infection to further enhance the anti-tumor response due to the presence of immunogenic peptides [25]. The FDA has approved three OV-based therapies for clinical use: RIGVIR, Oncorine, and T-VEC [26]. RIGVIR is an inartificial Enteric Cytopathogenic Human Orphan type 7 (ECHO-7) picornavirus that became the first OV to receive regulatory approval all around the world in 2004 [27, 28]. In 2005, Oncorine became the first recombinant oncolytic virus therapy approved by the Chinese state FDA and was used in combination with chemotherapy to treat head and neck cancer [29]. Talimogene laherparepvec (T-VEC), another OV therapy, received US FDA approval for the treatment of non-resectable metastatic melanoma in 2015 [30] and subsequently got approved for treatment of locally advanced or metastatic cutaneous melanoma in Europe, showing promising efficacy as a single agent and in combination with PD-1 inhibitor pembrolizumab [31]. Many additional oncolytic virus therapies based on Herpesvirus, Adenovirus, Vaccinia Virus, Coxsackievirus, and Vesicular stomatitis virus are in different phases of a clinical trial [32]. OV-based therapies have shown impressive results in clinical trials; but, the efficacy of this approach varies from person to person based on the immune system status, and these treatments are sometimes rapidly removed by the host immune system [25]. Furthermore, biosafety of OVs is a major issue in individuals having low immunity or patients who are on immune-suppressive drugs [33]. Adoptive T-cell therapies are another large, growing class of immunotherapies. There are various classes of adaptive cell therapies, including CAR-T cells, TCR-engineered T-cells, endogenous tumor-reactive T-cells, exogenously primed T-cells, and NK cell variants [34]. One of the most important and widely applicable modalities of adaptive T-cell therapies to emerge in recent times is CAR-T cell therapy. Generally, cellular therapies involve directing cells to recognize tumor-associated antigens (TAAs), with the goal of cells becoming activated upon recognition by immune cells and eliminating the tumor [35]. Adaptive cell therapy uses cancer antigen-specific T-cells such as tumor-infiltrating lymphocytes (TILs), engineered T cells, and peptide/cancer antigen-induced T-cells to treat cancer [36–38], leading to a long-lasting response in some patients with late-stage cancer. In 1988, TILs were the first cancer antigen-specific T-cell therapy used to treat melanoma [39, 40]. TIL-based therapy suppresses tumors in the circulation and tumor microenvironment while effectively killing cancer cells [41]. Despite the clinical benefits of TIL therapy, many challenges were associated with the therapy. TIL therapy requires the surgical removal of tumor tissues and the isolation and cultivation of TILs. Highly trained and skilled medical staff are needed to cultivate TILs; therefore, only a few medical centers can provide this therapy. To overcome this limitation of TILs, unmodified peptide-stimulated T cells and genetically engineered T cells such as T-cell receptor (TCR) were used in clinical trials, which achieved promising results [34, 36–38]. Peptide/cancer antigen-induced specific T cells are obtained by in vitro stimulation of peripheral blood mononuclear cells (PBMCs) collected from patients. Different cancer antigens and their derived human leukocyte antigen (HLA)-restricted epitopes facilitate the development of antigen-specific T cells for cancer treatment; however, cancer antigen-induced specific T cells are restricted by major histocompatibility complex (MHC) compatibility for targeting cancer antigens, the self-antigens expressed on both cancer and normal cells [42]. Stimulation of antigen-specific T cells for the long term may exhaust T cells and lead to shortened survival in vivo after infusion [43]. Genetically engineered TCRs are expressed on T cells to recognize cancer cells and kill them [34]. The gene transfer of TCR can be achieved in two ways: TCR from cancer-specific T cells can be derived from PBMCs or TILs, or TCRs can be produced via immunization of HLA-I/II transgenic mice with cancer antigen. The antigen-specific TCRs are cloned, transduced into the patient’s peripheral blood T cells via retroviral or lentiviral vectors, and amplified. TCRs derived from HLA-I/II transgenic mice usually have higher affinity than PBMC- or TIL-derived TCRs [44]. Various TCR-based T cell therapies have been in clinical trials: MART1 for treating metastatic melanoma [45], carcinoembryonic antigen (CEA) for treating colorectal cancer [46], NY-ESO-1 and gp100 for treating melanoma [47, 48], and MAGE-A3 for synovial sarcoma treatment [49]. Clinical trials data suggest that TCR therapy can be effective in some patients with haematological cancers like ALL but is more frequently applied in patients with melanoma [46, 48]. Furthermore, the TCR engineering method is laborious and time consuming, requiring HLA matching of patients and TCR clones and the presentation of tumor-antigen on MHC that is generally down-regulated in tumor cells, restricting its application. Antibody–drug conjugates (ADCs) have surfaced as a promising strategy for targeted cancer therapy [50]. They work by tethering a chosen therapeutic agent to an antibody specific to an antigen overexpressed on cancer cells. With several ADCs already receiving clinical approval, this innovative approach is actively broadening the spectrum of precision oncology [51]. Cytokines are another integral component of the immune response to cancer. These small proteins play critical roles in regulating immune and inflammatory responses, facilitating communication between cells. Two types of cytokines, interferons and interleukins, have been harnessed for cancer therapy due to their ability to inhibit tumor growth and stimulate the immune system. For instance, high-dose Interleukin-2 (IL-2) has been used in the treatment of certain types of kidney cancer and melanoma, albeit with variable success and significant toxicity [52]. Immunomodulators are agents that adjust immune responses, either by enhancing or suppressing them, and they have become key players in the arsenal of cancer treatments. Immunomodulatory drugs (IMiDs), like thalidomide and its analogs (lenalidomide and pomalidomide), have been particularly successful in the treatment of multiple myeloma and certain myelodysplastic syndromes [53]. They work by modulating the tumor microenvironment and enhancing the body's immune response against cancer cells. It's important to note that while these therapeutic strategies have significantly advanced our ability to treat various cancers, they are not without their limitations. Some patients may not respond to these therapies, and others may experience significant side effects. Additionally, the potential for resistance to these therapies necessitates ongoing research to identify new targets and develop new treatment strategies. The recent success of CAR-T cell therapy in hematological tumors has spurred a lot of interest in this field, and rapid progress has occurred in the last 5 years, as evidenced by the approval of multiple CAR-based therapies targeting CD19 and B cell maturation antigen (BCMA) [88, 89, 133]. CAR-T cell therapy has been especially transformative in the management of relapsed and refractory malignancies, where traditional first and second-line therapies have proven ineffective.

## The emergence of CAR-T cell therapy

Chimeric antigen receptors (CARs) are genetically engineered synthetic receptors that function as immune effector cells, similar to lymphocyte T cells. These receptors recognize cells expressing specific target antigens and eliminate them [54, 55]. CAR-T cells can be categorized into two types, based on the origin of the cells used: autologous and allogenic. Autologous CAR-T cells are derived from the patient's own blood. In this process, blood is drawn from the patient and then subjected to a procedure known as leukapheresis, which separates out the white blood cells, including T cells. These T cells are then genetically modified in the laboratory to express a specific CAR that enables them to recognize and destroy cancer cells. Once the modification is successful, these engineered T cells are expanded in number and then reinfused back into the patient. Allogenic CAR-T cells, on the other hand, originate from the blood of a healthy donor. In this case, T cells are isolated from the donor's blood and then bioengineered to express the desired CAR. These engineered cells are subsequently expanded and infused into the patient. This approach, while presenting its own set of challenges such as potential graft-versus-host disease, offers the possibility of an 'off-the-shelf' treatment, providing readily available therapeutic options for patients in need [56]. The binding of CAR to cell surface-expressed target antigen is independent of the major histocompatibility complex (MHC) receptor, resulting in strong activation of CAR-T cells and potent anti-tumor response [57], which is a main advantage of CAR-T cell over other forms of adaptive T cell therapy.

**Fig. 1:**
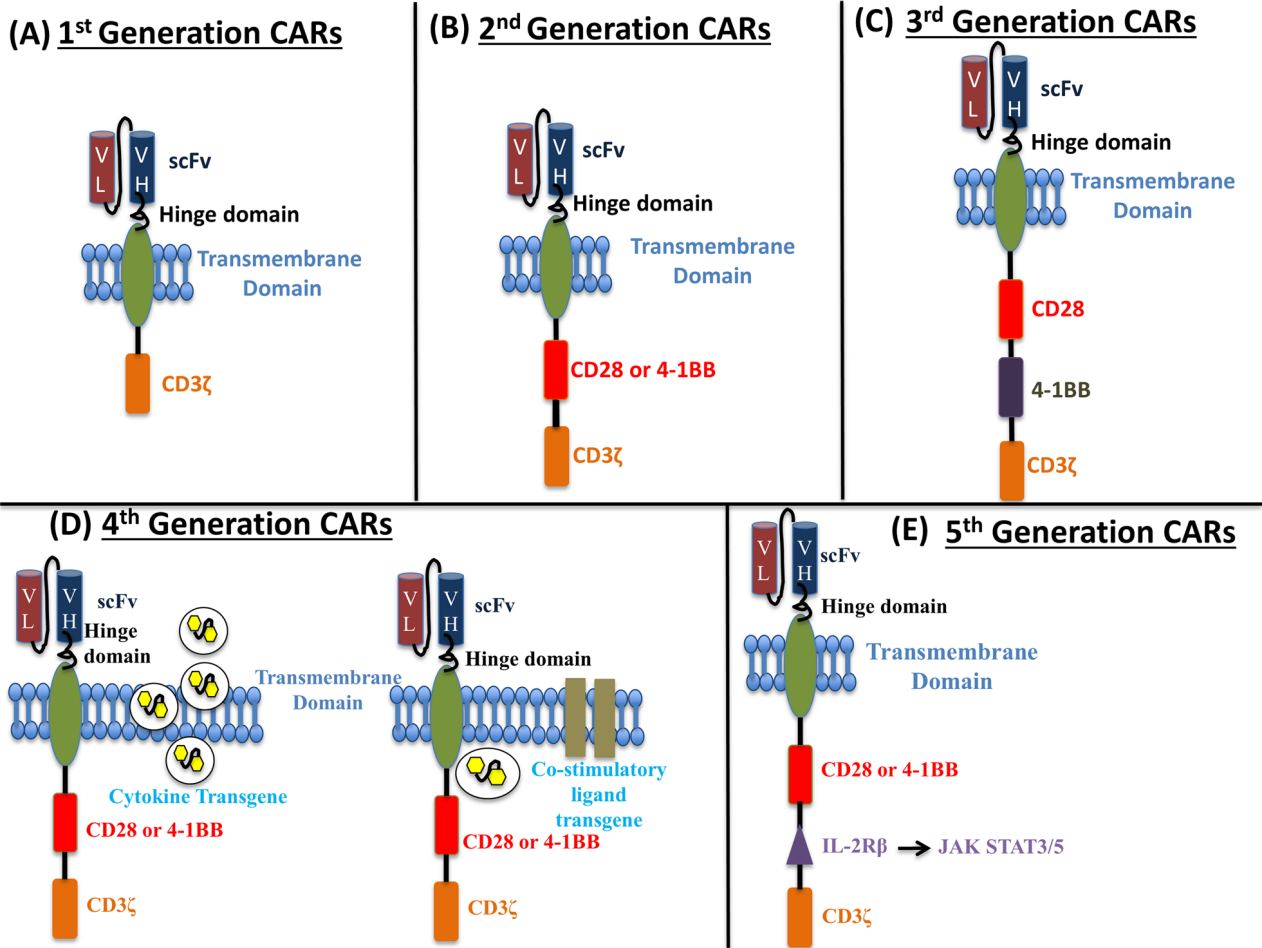
Structural design of different generations of CARs. **A** First-generation CARs with ScFv, Hinge, Transmembrane, and CD3ζ domains. **B** Second-generation CARs with all first-generation domains and an additional CD28/4-1BB costimulatory domain. **C** Third-generation CARs with all first-generation domains and two additional costimulatory domains (CD28 and 4-1BB). **D** Two different types of fourth-generation CARs with additional cytokine and co-stimulatory ligand domains to address challenges of tumor microenvironments. **E** Fifth-generation CARs with one intracellular domain more than the fourth-generation CAR-T cells, which include truncated intracellular domains of cytokine receptors (e.g., IL-2R chain fragment), with an additional domain for binding transcription factors such as STAT-3/5. CARs, Chimeric antigen receptors; ScFv, single-chain variable fragment

As shown in Fig. 1b, the second-generation CAR typically consists of four domains: Antigen-binding domain, Hinge region, Transmembrane domain, and Intracellular T-cell signaling domain. The antigen-binding domain is an extracellular domain that interacts with the target antigen [58, 59]. The domain is made up of monoclonal antibodies’ variable heavy (VH) and variable light (VL) chains, which are connected with short linker peptides of serine-glycine or glutamine-lysine to form a single chain variable fragment (scFv) [60, 61]. The hinge or spacer region is a tiny domain that connects the antigen binding domain and the outer membrane of the CAR-T cell [62]. The hinge provides flexibility to the receptor. The spacer’s length depends on the antigen epitope; typically, long spacers are used to increase the flexibility of receptors and to provide better attachment to membrane-proximal epitopes. In contrast, short spacers bind better to membrane-distal epitopes [63–65]. The optimal spacer length generally depends on the target epitope position. The hinge domain plays a crucial role in the overall performance of the CAR-T cells. The transmembrane domain is between the hinge region and the intracellular signaling domain [66] and is derived from natural proteins such as CD3ζ, CD4, CD8, or CD28 [67]. The transmembrane domain's primary function is to anchor the CAR to the T cell’s membrane, and it is also relevant for the CAR-T cell's effector function [68, 69]. When an antigen binds to the antigen binding domain, CARs come close and cluster together, giving an activation signal to the intracellular T cell signaling domain, and the domain transmits this signal to the inside of the cell [57].

CAR-T cell therapy is a personalized, living drug for those with cancer and has shown promise for treating hematological malignancies. CAR-T cell therapy is a promising approach for cancer treatment compared to conventional therapeutic approaches in terms of survival and cancer recurrence [70]. Other forms of adoptive immunotherapies [71] include T-cell receptor (TCR) therapy and tumor-infiltrating lymphocytes (TIL) therapy which require MHC molecule activation, but CAR-T cells can be activated independently [72, 73]. TCR therapy is similar to CAR T-cell therapy in that it involves the genetic modification of a patient's T-cells. However, TCR therapy modifies the T-cells to express a specific T-cell receptor that can recognize a particular antigen presented by cancer cells. Unlike CARs, TCRs can recognize antigens from within the cell that are presented on the cell surface by the major histocompatibility complex (MHC). This allows TCR therapy to target a broader range of antigens, including those derived from proteins inside the cancer cell [48]. However, TCR therapy is MHC-dependent, which means its effectiveness can be affected by changes in MHC expression or antigen processing in the cancer cells.

TIL therapy is another type of adoptive cell therapy that doesn't involve genetic modification of T-cells [74]. In TIL therapy, lymphocytes are extracted from a patient's tumor, expanded in the laboratory, and then reinfused into the patient. These lymphocytes, having been naturally present within the tumor, are already primed to recognize and attack the patient's cancer cells. However, the success of TIL therapy can be influenced by the heterogeneity of tumors and the variable presence of TILs within different tumors. Overall, while CAR T-cell, TCR, and TIL therapies all involve the use of a patient's immune cells to fight cancer, they differ in their strategies for recognizing and targeting cancer cells. Multiple generations of CAR-T cells have been developed. Still, typically, each CAR consists of an antigen recognition domain linked to a T cell co-stimulatory domain and an activation domain [61]. New variants continue to evolve to overcome challenges imposed by tumor microenvironments in different cancers. CARs have been divided into different generations according to their domain configuration, as discussed in the following section.

## Different generations of CAR-T cells

In 1989–1993, Zelig Eshhar and Gideon Gross developed the first-generation CAR-T cells at the Weizmann Institute of Science in Israel. These CARs directed T cells to kill tumor cells in vitro and were comprised of the TCR α and β chains fused to the variable region of an antibody's light and heavy chain (Fig. 1a) [75]. The first-generation CARs were later combined into single molecules with intracellular domains of T cell co-receptor and extracellular domains of ITAM-containing immunoreceptors [76, 77]. However, first-generation CAR-T cells were clinically safe but ineffective due to their inability to produce sufficient costimulatory signals for effective target cell killing. As a result, second-generation CAR-T cells were developed to provide better co-stimulation and have recently been approved for the treatment of many forms of hematological malignancies. Initially, co-stimulation of T cells was achieved through the CD28 ligand, but 4-1BB, a TNFRSF member, was later incorporated into some second-generation CARs (Fig. 1b) [78–82]. Third-generation CARs have two co-stimulatory domains, most commonly CD28 and 4-1BB, and confer even greater anti-tumor potency on human CAR-transduced T-lymphocytes (Fig. 1c). Compared to their second-generation counterparts, third-generation CARs were found to have higher levels of phosphorylation status upon binding to their target antigen, indicating greater intracellular signaling [83, 84]. The enhanced phosphorylation and increased intracellular signaling allow the third-generation CARs to have more significant expansion and support differentiation into memory subsets [83, 85]. However, the third-generation CAR-T cells have been linked to a greater risk of immune-related adverse effects due to the greater activation of CAR-T cells and have come under increased scrutiny due to safety concerns. A fourth-generation of CAR-transduced T cells, termed T-cells redirected for universal cytokine killing (TRUCKs) are engineered to release a transgenic product, commonly a pro-inflammatory cytokine such as IL-12, once their CAR is activated by the target antigen (Fig. 1d) [86]. This results in further enhancement of T-cell proliferation and function and, more importantly, in the recruitment of innate immune effectors specifically to the tumor microenvironment, thus inducing superior tumor killing while limiting cytokine-mediated systemic toxicity [87]. Fifth-generation CARs are currently being developed to make CAR-T cells safer and more productive by incorporating one more intracellular domain than their predecessors and adding drug-dependent OFF-switches or ON-switches leading to CAR depletion or activation, respectively [88].

While there have been concerns over the safety of third-generation CAR-T cells due to the greater activation of CAR-T cells, the favorable properties of third-generation and fourth-generation CAR-T cells make them a promising alternative to earlier generations. The ongoing development of fifth-generation CARs aims to address challenges related to off-target and off-tumor activity and further enhance the effectiveness and safety of CAR-T cell therapy.

## CAR-T cell clinical application

### CAR-T cell therapy in blood cancers

In the last decade, immunotherapy approaches, including CAR T-cell therapy, have significantly improved the clinical prognosis of patients with hematological malignancies. Indeed, CAR-T cell therapy has portended promising clinical outcomes in the treatment of lymphomas, multiple myeloma, and leukemia [55, 89–92]. Hence, in the last 5 years, the Food and Drug Administration (FDA) has approved several CAR-T cell products for the treatment of acute lymphoblastic leukemia, chronic lymphocytic leukemia, multiple myeloma, and different forms of lymphomas (including diffuse large B-cell lymphoma [DLBCL], primary mediastinal B-cell lymphoma, and transformed lymphoma) [92, 93]. For instance, the outcome of CAR-T cell therapy was particularly remarkable in patients with aggressive forms of blood malignancies that did not respond or relapsed upon multiple chemotherapies [78, 79].

#### Leukemia

Acute lymphoblastic leukemia (ALL) is a heterogenous malignancy in children and adults and is characterized by abnormal modification and proliferation of lymphoid progenitors. Although chemotherapy and hematopoietic stem-cell transplantation (HSCT) improved patients’ prognosis, some patients with ALL showed a refractory disease or experienced relapse, emphasizing the need for more efficient therapies [92, 94]. In the last decade, the efficacy of CAR-T cell therapy in ALL has been reported in several clinical trials [55, 91, 92, 95–97]. Some studies opted for a pre-conditioning chemotherapy including fludarabine and cyclophosphamide followed by the infusion of autologous CD19-specific CAR T-cells (CTL019) in children and adult patients suffering from refractory/relapsed (r/r) ALL. These studies found that a durable complete remission (CR) rate was achieved in 70–90% of the treated patients [55, 91, 97]. CAR T-cell therapy has shown a particular clinical benefit in patients suffering from r/r B-precursor ALL with poor prognosis [10, 92, 98, 99]. A phase 1 long-term cohort study conducted at the Memorial Sloan Kettering Cancer Center in 53 adult patients (median age, 44 y) with relapsed B-ALL showed that autologous CAR-T cells therapy led to a complete remission (CR) rate of 83% with a median overall survival (OS) of 12.9 months (95% CI 8.7–23.4) [100]. Interestingly, in a phase 2 multicenter study, Maude et al. demonstrated that children and young adults with recurrent or refractory B-ALL treated with the CD-19-targeted CAR-T cell product tisagenlecleucel, showed a high overall remission rate of 82% with a durable response up to 12 months (76% OS and 50% event-free survival at 12 months) [10]. This study led to the FDA approval of tisagenlecleucel (Tisa-cel or Kymriah^®^) therapy for high-risk relapsed or refractory (r/r) B-ALL in pediatric and young adult patients (age, up to 25 years) [101, 102].

More recently, a phase 1 long-term study in children and young adults (age, 3–30 years) with B-ALL reported a CR rate in 62% of patients after treatment with autologous CD19-CAR T-cells [103]. Recently, Marianna Sabatino et al. conducted a phase 1/2 cohort study (ZUMA-3) in adults with B-ALL to test the safety and clinical efficacy of KTE-X19, an autologous anti-CD19 CAR-T cell product whose manufacturing process enables the elimination of malignant cells [104]. The ZUMA-3 phase 1 study demonstrated that a single infusion at 1 × 10^6^ per kg of KTE-X19 was associated with tolerable side effects and a significant CR rate (83%) in adults with r/r B-ALL [105]. Interestingly, the same group has recently revealed the results of the ZUMA-3 phase 2 multicenter study, which demonstrated in a larger population of r/r B-ALL adult patients that KTE-X19 conferred a significant rate of long-term CR (71%), a median OS of more than 18 months, and undetectable minimal residual disease in 97% of responder patients [106]. KT-X19 (brexucabtagene autoleucel, Tecartus™) is FDA approved for the treatment of adults with relapsed or refractory mantle cell lymphoma (MCL) [107]. On the basis of the ZUMA-3 phase 1 and 2 findings, the FDA approved the use of KT-X19 to treat r/r B-cell precursor ALL in October 2021 [107]. Nevertheless, although anti-CD19 CAR-T cell therapy showed the most prominent clinical outcome in B-ALL, it was demonstrated that the tumor cells could escape this immunotherapeutic approach. Therefore, two potential targets, CD20 and CD22, are being tested in r/r B-ALL [98, 108].

Chronic lymphocytic leukemia (CLL) is the predominant type of leukemia in adulthood. It is a heterogeneous malignancy with variable OS rates and short progression-free expectancy [109, 110]. In addition to chemotherapy, allogeneic HSCT is commonly used for the treatment of patients with CLL. Nevertheless, many patients’ disease relapsed or was irresponsive to conventional chemotherapy; some patients are not eligible for HSCT therapy. Hence, CLL remains mostly irremediable, and patients with r/r CLL have a poor clinical prognosis [111]. Treating B-cell malignancies such as CLL by using anti-CD19 CAR T-cells seems to be a rational and effective therapeutic approach. Indeed, several studies demonstrated that patients with r/r CLL treated with CD-19-targeting CAR T-cells showed a persistent and long-term CR rate [112–116]. In a clinical trial of 18 adults with CD19 + CLL who were heavily pretreated and had recurrent or sustained disease, Porter and colleagues showed that 8 (57%) of the patients responded to autologous CD19 CAR-T cells therapy [113]. Among these patients, 4 experienced durable CR, and the remaining experienced partial remission (PR). Interestingly, none of the patients with CR experienced relapse over a follow-up period of 49 months [113]. Similarly, in a phase I/II clinical trial, Turtle et al. showed that CD-19 CAR-T cells therapy was clinically successful in 71% of patients experiencing high-risk CLL, which progressed after ibrutinib treatment [116], a Bruton’s tyrosine kinase inhibitor initially approved as first-line therapy for r/r CLL [117]. Additionally, molecular CR was associated with progression-free survival up to 6.6 months after CAR-T cell therapy [116]. More recently, a phase II clinical trial investigated the long-term efficacy and the optimal dose of autologous CD19 CAR-T cells in patients with relapsed or persistent CD19-positive CLL. The findings of this study revealed that overall response was observed in 44% of patients and that CR was most common in the group that received the higher dose of CAR-T cells. Nevertheless, regardless of CAR-T cells dose, progression-free survival was significantly longer (median, 40.2 months) in patients who experienced CR than in those who did not [112]. However, despite the promising clinical outcome of CAR-T cells for r/r CLL, the efficiency of the production process of autologous CAR T-cells is debatable, specifically related to the viability of the T cells isolated from the heavily treated patients and the long manufacturing duration [118]. Moreover, further investigations on larger population sizes are needed to confirm the efficacy and safety of CAR-T cells in CLL and to characterize how the molecular diversity of the disease would affect the clinical effect of this immunotherapy. Currently no CAR-T cell construct is approved for CLL treatment.

#### Lymphomas

CAR-T cells are one of the most advanced immunotherapeutic approaches for patients with r/r B cell non-Hodgkin lymphoma (B-NHL) that resisted multiple chemotherapies and/or HSCT [89, 92, 116, 119–123]. In a phase I/II clinical trial, Turtle and colleagues evaluated the efficiency and safety of adoptive CD19 CAR-T cell therapy in patients with advanced CD19 + B cell malignancies, including NHL. They observed that the overall response rate for patients who received CAR-T cells after lymphodepletion was 84%, in which 47% of the patients experienced CR [124]. Allogeneic anti-CD19 CAR-T cells have been tested in patients with B-cell malignancies, including ALL, CLL, and B-NHL, for whom allogeneic HSCT was unsuccessful [125]. Interestingly, the 20 treated patients did not receive any priming lymphodepletion, and 40% of them experienced partial (10%) or complete (30%) remission following infusion of allogeneic CAR-T cells [125]. Wang and colleagues investigated the potential of autologous CD19 CAR-T cell therapy in improving the remission rates in patients with B-NHL who received autologous HSCT. Ex vivo-expanded autologous CD19 CAR-T cells were infused into 8 patients 2 days following HSCT. The observed overall response was 63% (38% CR and 25% PR) [120]. However, notable response rates were observed in patients with large B-cell lymphomas treated with CAR T-cell after failure of the disease to respond to several standard lines of chemotherapy [89, 123, 126, 127].

Several CAR-T cell products are FDA-approved for the treatment of lymphoma. Yescarta^®^ (Axicabtagene ciloleucel, Axi-cel) is a CD19-directed CAR-T cell product designed by researchers [114, 128, 129] and manufactured by Kite pharma, Inc. Yescarta^®^ received its first FDA approval in 2017 to treat patients with follicular lymphoma (FL) who did not respond or relapsed after at least two previous lines of systemic chemotherapy [130]. FDA approval was based on the results of a phase 1/2 clinical trial (ZUMA-1) of 101 patients with NHLs (77 cases with diffuse large B-cell lymphoma (DLBCL) and 24 cases with primary mediastinal large B-cell lymphoma (PMBCL). In this study, Locke and colleagues demonstrated that overall survival was observed in 50% of the treated patients, with a rate of 41% progression-free survival for up to 2 years [131]. More recently, the same group conducted a phase 3 clinical trial to check the efficacy and safety of using Yescarta^®^ as second-line therapy for patients with large B-cell lymphoma whose disease did not respond or had relapsed 12 months after first-line chemoimmunotherapy [127]. Their findings suggested that treatment with Axi-cel significantly improved the clinical outcome of the patients. For instance, an overall response (OR) of 83% with 50% CR was observed in the Axi-cel patients compared to 50% OR, including 32% CR, in the standard-care group [127]. Also, event-free survival was significantly higher in the Axi-cel therapy group [8.3 months (95% CI 4·5–15·8) vs. 2 months (1·6–2·8)]. Based on these findings, in 2022, Yescarta^®^ was granted FDA approval to be used as second-line therapy of adult large B-cell lymphoma not responding to first-line chemoimmunotherapy or relapsing within 1 year of first-line chemoimmunotherapy [130].

Tisagenlecleucel (Tisa-cel or Kymriah^®^), an autologous CD19-targeted CAR-T cell product, is FDA approved for treating patients with r/r DLBCL that fails at least two previous lines of systemic therapy [101]. This approval was issued based on the findings of a phase 2 study. This pivotal JULIET study showed that an ORR of 52% with 40% CR was observed in refractory DLBCL patients who were treated with a single infusion of Tisagenlecleucel [33]. As mentioned above, Kymriah^®^ is also approved for treating B-cell leukemia in children and adults up to 25 years old. Very recently, Kymriah^®^ got a new FDA approval for adult patients with r/r follicular lymphoma after two or more lines of standard therapy [101, 102]. This approval was generated upon publication of the ELARA trial results, which reported that CR was observed in 68% of patients receiving Kymriah^*®*^ along with a sustained clinical response (85% of cases at 12 months) and a tolerable safety profile [132]*.* However, tisagenlecleucel did not exhibit any advantage compared to standard-care second-line therapies in patients with refractory or early-relapsed aggressive lymphoma (within 12 months) [133].

In a phase 1/2 study by Abramson et al. patients with r/r large B-cell lymphomas (including DLBCL, DLBCL transformed from indolent lymphoma, and PMBCL) treated with Lisocabtagene maraleucel (Liso-cel; Breyanzi^*®*^), a second-generation anti-CD19 CAR-T cell product, experienced 73% ORR with 53% CR [89]. These findings led to FDA approval of Liso-cel for the treatment of r/r DLBCL, PMBCL, and follicular lymphoma grade 3B after a minimum of two previous standard treatments [134]. Additionally, a recent phase 3 clinical trial (TRANSFORM study) showed that Liso-cel therapy significantly improved the median event-free survival (10·1 month in the Liso-cel-treated group vs. 2·3 months in the standard-care group) in patients with early relapse (less than 1 year) or refractory large B-cell lymphoma, and the authors suggested using Liso-cel as a new second-line therapy for this category of patients [135].

#### Mantle cell lymphoma

Mantle cell lymphoma (MCL) is a rare, yet aggressive type of B-cell lymphoma with a poor prognosis despite available therapeutic strategies. CAR-T cell therapies have demonstrated efficacy in high-risk MCL [136, 137]. Brexucabtagene autoleucel (Tecartus^®^) is the only FDA-approved CAR T-cell therapy for adult patients with r/r mantle cell lymphoma [107, 138, 139]. The FDA approval was based on findings obtained during the ZUMA-2 phase 2 multicenter clinical trial studying the efficacy and safety of Tecartus^®^ in adult patients with relapsed or refractory MCL who failed prior therapies, including chemotherapy, anti-CD20 antibody, and a Bruton’s tyrosine kinase inhibitor. This study showed that 93% of the patients treated with Tecartus^®^ showed an objective response, with 67% cases with CR. Moreover, the patients’ follow-up at 12 months achieved 61% PFS and 83% OS, indicating a durable response upon CAR T-cells therapy [140].

Albeit the clinical efficacy of CD-19-specific CAR T cells is proven in aggressive B-cell lymphoma, long-term disease control failed in many patients [92, 141]. Hence, there is an unmet need for strategies to improve CAR-T cell therapeutic approach. Promising clinical outcome was observed with third-generation CAR T cells targeting CD20 in B-cell non-Hodgkin lymphomas (B-NHL) [142]. In a pilot, clinical trial, Till and colleagues investigated the efficacy of anti-CD20 CAR T-cells in a pilot study of 3 patients with relapsed NHL. Two patients experienced complete remission without progression for up to 24 months; yet, the third patient showed partial remission, with disease relapse 1 year after treatment [142]. Recently, Zhang Y. et al. published the data of a phase I/II study in which they checked the effect of targeting concomitantly or separately both CD19 and CD20 using tandem CD19/CD20 CAR-T cells (TanCAR7 T cells) for the treatment of relapse/refractory NHL following chemotherapy lymphodepletion [143]. The maximum overall response was 78% (95% CI 68–86); 70% (95% CI 59–79) of the cases achieved a CR, and the remaining exerted a PR. Moreover, 60% of the patients were disease free amid a follow-up beyond 2 years [143].

#### Multiple myeloma

Multiple myeloma (MM) is hematopoietic cancer that arises from mutated plasma cells, called myeloma, which proliferate uncontrollably within the bone marrow [92, 144]. Although multiple myeloma is rare cancer, it has been recently reported that its global incidence is rising [145]. Despite the advances in the diagnosis and therapeutic management of multiple myeloma, this cancer remains incurable [144].

FDA-approved investigational CAR-T cell therapies targeting one or more antigens expressed by myeloma cells are considered promising therapeutic approaches for MM [146]. The CD138 antigen, highly expressed in myeloma cells, is an important diagnostic and therapeutic biomarker [144, 147]. A pilot study conducted by Guo et al. reported that patients with advanced MM tolerated and favorably responded to autologous anti-CD138 CAR T-cell therapy; Out of 5 patients, 4 presented stable disease above 3 months and 1 patient showed a significant decrease in the percentage of peripheral blood myeloma cells (from 10.5% to less than 3%) [148]. Another hallmark of a differentiated plasma cell is the B-cell maturation antigen (BCMA) [92, 147, 149]. Several clinical trials reported the feasibility, safety, and efficiency of anti-BCMA CAR T cells in MM patients [150–152]. Currently, there are two FDA approved second-generation autologous anti-BCMA CAR-T cell constructs for the treatment of relapsed/refractory in patients with MM that failed four or more prior therapies; Idecabtagene vicleucel (Ide-Cel; Abecma^®^) is the first CAR T-cell product to obtain FDA approval (March, 2021) to treat r/r MM patients [153, 154] (Table 1). Abecma^®^ gained its approval based on the results of a phase 2 study (KarMMa study) conducted on 140 MM patients whose disease was irresponsive or relapsed after at least 3 prior anti-myeloma treatment regimens [155]. This study showed an overall response in 73% of the heavily pre-treated patients with r/r MM, with a CR in 33% of the cases [155]. In February 2022, the FDA approved the second BCMA-directed CAR-T cell therapy for r/r MM: ciltacabtagene autoleucel (Cilta-Cel; Carvykti^®^) [156]. The approval of Carvykti^®^ was based on the findings of the CARTITUDE-1 study, a phase 1b/2 trial showing 98% ORR and 80% stringent CR in 97 CAR-T cell-treated patients exhibiting r/r MM. Additionally, 12 months after Carvytki^®^ infusion, the OS and PFS were 89% and 77%, respectively [157].

**Table 1:** US FDA-approved CAR-T cell therapies

Generic name	Trade name	Target antigen	Indication	Date of FDA approval
Axicabtagene ciloleucel	Yescarta^®^	CD19	Large B cell lymphoma	October 2017
			Follicular lymphoma	March 2021
Tisagenlecleucel	Kymriah^®^	CD19	Acute lymphoblastic leukemia	August 2017
			Large B cell lymphoma	May 2018
Brexucabtagene autoleucel	Tecartus^®^	CD19	Mantle cell lymphoma	July 2020
Liscobtagene maraleucel	Breyanzi^®^	CD19	Large B cell lymphoma	February 2021
Idecabtagene vicleucel	Abecma^®^	BCMA	Multiple myeloma	March 2021
Ciltacabtagene autoleucel	Carvykti^®^	BCMA	Multiple myeloma	February 2022

On the basis of pre-clinical data, many researchers suggested the potential benefit of using CAR T-cells targeting other plasma cells biomarkers such as the G-protein-coupled receptor, class C group 5 member D (GPRC5D), SLAM Family Member 7 (SLAMF7) and integrin ß1 [158–163]. In the phase 1 clinical trial, Mailankody and colleagues recently observed an overall response rate of 71% in MM patients treated with GPRC5D-directed CAR-T cells [164]. Interestingly, this study showed a response in 6 (75%) of 8 patients whose disease relapsed after BCMA CAR-T cell therapy, emphasizing the importance of GPRC5D as an immunotherapeutic target in MM [164]. Further, researchers and clinicians are also exploring the potential of dual-targeted CAR-T cells, such as BCMA/GPRC5D-directed CAR-T cells, to improve the efficacy of this approach in patients with MM [165, 166].

#### Novel CAR-T cell targets and adjuvant therapies in development

The most common CAR-T cell constructs used for B-cell malignancies target CD19. However, additional tumor targets such as CD20, CD30, CD38, and CD138 have also been investigated [148, 167–169]. Similarly, new targets have been explored for treating multiple myeloma [146, 147]. Moreover, substantial efforts are made to assess the potential advantage of combining CAR-T cells with standard systemic anti-cancer therapies to improve clinical prognosis in patients with hematological malignancies [170].

Some of the most challenging drawbacks of CAR-T cell therapy are its short- and long-term adverse events (AEs). The most frequently observed AE of CAR-T cells is cytokine release syndrome (CRS), characterized by an upregulation of systemic inflammatory cytokines. The clinical presentation of CRS varies from mild (high fever, hypotension) to life-threatening toxicity (multi-organ failure, neurotoxicity, seizures, and coma) [92, 146, 171]. After CAR-T cell infusion, some patients also have decreased blood counts leading to reduced immunity and increased infections [146]. Managing these AEs includes prophylactic antimicrobial treatments to prevent severe infection and antibody therapy for patients undergoing CAR-T cell therapy to reduce the risk of related neurological toxicity [159].

Several studies are focused on developing strategies to reduce the toxicity of CAR-T cell therapy. For instance, in a recent clinical trial, systemic corticosteroids were used as a prophylactic treatment in patients with large B‐cell lymphoma treated with axicabtagene ciloleucel (Axi-cel) CAR-T cells [172]. This study showed that the use of prophylactic corticosteroids prevented grade 3 toxicity, hindered the onset of CRS, and had no affect on the clinical efficacy of Axi-cel [172]. Additionally, antibodies targeting the Interleukin-6 receptor (anti-IL6R), such as tocilizumab, successfully counteracted CRS symptoms in CAR-T cell-treated patients with B-ALL [55, 96]. The cytogenetic and molecular heterogeneity of hematopoietic cancers plays an essential role in the clinical outcome of CAR-T cell therapy, so the selection criteria for the enrolled patients substantially affects the variation of the evaluated clinical endpoints. Therefore, exploring more specific tumor cell targets as well as the development of bispecific CAR-T cells is of the utmost importance to achieve an optimal and personalized application of CAR-T cells in blood cancers. Bi-specific CAR T-cells are a promising new approach that has been designed to address the issue of tumor antigen heterogeneity, which can limit the efficacy of traditional CAR T-cell therapies [173]. These bi-specific CAR T-cells are engineered to express two different CARs, enabling them to recognize and target two different tumor-associated antigens simultaneously [174]. By doing this, they are capable of recognizing a broader range of cancer cells within the same tumor, thereby improving their anti-tumor efficacy. Moreover, bi-specific CAR T-cells could potentially delay or prevent the development of tumor antigen escape, a common resistance mechanism where cancer cells evade CAR T-cell recognition by down-regulating or mutating the target antigen [175]. These advancements underline the ongoing efforts to improve the safety and effectiveness of CAR T-cell therapy, and it's our hope that they will provide a solid foundation for future developments in this exciting field.

### CAR-T cell therapy in solid cancers

Despite the success of CAR-T cell therapy in relapsed or refractory hematological malignancies, it still faces a challenge for the treatment of solid tumors. Solid tumors have a unique TME that is characterized by abnormal vasculature, dense extracellular matrix, interstitial fluid pressure, hypoxia, and the presence of immunosuppressive cells, all of which are contributing factors in preventing the infiltration of CAR-T cells [176]. Apart from the complex tumor niche, a significant hurdle in solid tumor CAR-T therapy is target antigen heterogeneity. Unlike hematological malignancies such as ALL or CLL, in which tumor cells express tumor-specific antigens, solid tumors rarely express one tumor-specific antigen [177]. Solid tumors commonly contain tumor-associated antigens (TAAs), which are self-antigens that are abnormally expressed in tumors and expressed at low levels in a subset of normal host cells [178]. Overall, 22 TAAs are being investigated in patients with solid tumors in ongoing clinical trials (reviewed in [179]). Of these, the most frequently targeted that have been characterized include mesothelin [180], mucin-1 (MUC-1) [181], EGFR variant III [182], carcinoembryonic antigen (CEA) [183], CA-IX [184], GD2 [185], ERBB2 [186, 187], and prostate-specific membrane antigen (PSMA) [188] (Table 2).

**Table 2:** Major ongoing clinical trials of CAR-T cell therapy in different cancers

Condition	Intervention	Phase	Clinical trial identifier	Estimated completion date	Sponsors/site	Status	Source
Breast cancer	CEA	Phase I/II	NCT04348643	April 30, 2023	Chongqing Precision Biotech Co., Ltd	Recruiting	Clinicaltrials.gov, NIH, U.S. National Library of Medicine
	Multi-4SCAR T	Phase I/II	NCT04430595	December 31, 2023	Shenzhen Geno-Immune Medical Institute	Recruiting	Clinicaltrials.gov, NIH, U.S. National Library of Medicine
					The Seventh Affiliated Hospital of Sun Yat-sen University		
	Anti-hCD70	Phase I/II	NCT02830724	January 1, 2028	National Cancer Institute (NCI)	Recruiting	Clinicaltrials.gov, NIH, U.S. National Library of Medicine
	cMET RNA	Phase I	NCT01837602	August 13, 2018 (Completed)	University of Pennsylvania	Completed	Clinicaltrials.gov, NIH, U.S. National Library of Medicine
	huMNC2-CAR44	Phase I	NCT04020575	January 15, 2035	Minerva Biotechnologies Corporation	Recruiting	Clinicaltrials.gov, NIH, U.S. National Library of Medicine
					City of Hope Medical Centre		
	EGFR/B7H3	Early Phase I	NCT05341492	May 1, 2025	Second Affiliated Hospital of Guangzhou Medical University	Recruiting	Clinicaltrials.gov, NIH, U.S. National Library of Medicine
	HER2-BPX-603	Phase I/II	NCT04650451	January 2, 2027	Bellicum Pharmaceuticals	Recruiting	Clinicaltrials.gov, NIH, U.S. National Library of Medicine
	Anti-CD133	Phase I/II	NCT02541370	June 2019	Chinese PLA General Hospital	Completed	Clinicaltrials.gov, NIH, U.S. National Library of Medicine
Multiple myeloma	CD138	Phase I/II	NCT01886976	June 2016	Chinese PLA General Hospital	Completed	Clinicaltrials.gov, NIH, U.S. National Library of Medicine
	BCMA	Early Phase I	NCT05430945	June 20, 2025	Zhejiang University	Recruiting	Clinicaltrials.gov, NIH, U.S. National Library of Medicine
					Yake Biotechnology Ltd		
	CS1	Early Phase I	NCT04541368	December 31, 2026	Zhejiang University	Recruiting	Clinicaltrials.gov, NIH, U.S. National Library of Medicine
					Yake Biotechnology Ltd		
	BCMA	Phase I/II	NCT04271644	July 1, 2023	Chongqing Precision Biotech Co., Ltd	Recruiting	Clinicaltrials.gov, NIH, U.S. National Library of Medicine
	ALLO-715	Phase I	NCT04093596	December 2027	Allogene Therapeutics	Recruiting	Clinicaltrials.gov, NIH, U.S. National Library of Medicine
	Anti-CD19/BCMA	Phase I	NCT03706547	December 2021	Peng Liu	Unknown	Clinicaltrials.gov, NIH, U.S. National Library of Medicine
					Hrain Biotechnology		
					Shanghai East Hospital		
	Anti-BCMA/GPRC5D	Phase II	NCT05509530	May 1, 2025	Xuzhou Medical University	Recruiting	Clinicaltrials.gov, NIH, U.S. National Library of Medicine
	BCMA	Phase I/II	NCT03548207	August 23, 2022	Janssen Research and Development, LLC	Completed	Clinicaltrials.gov, NIH, U.S. National Library of Medicine
	BCMA	Phase I	NCT04706936	April 2024	Second Affiliated Hospital, School of Medicine, Zhejiang University	Recruiting	Clinicaltrials.gov, NIH, U.S. National Library of Medicine
					Carbiogene Therapeutics Co. Ltd		
	CS1 (UCART)	Phase I	NCT04142619	December 11, 2024	Cellectis S.A	Recruiting	Clinicaltrials.gov, NIH, U.S. National Library of Medicine
Glioblastoma	CD147	Phase I	NCT04045847	May 30, 2022	Xijing Hospital		Clinicaltrials.gov, NIH, U.S. National Library of Medicine
	IL13Rα2	Phase I	NCT04003649	December 1, 2022	City of Hope Medical Center		Clinicaltrials.gov, NIH, U.S. National Library of Medicine
					National Cancer Institute (NCI)		
	EphA2	Phase I	NCT03423992	January 30, 2023	Xuanwu Hospital, Beijing	Recruiting	Clinicaltrials.gov, NIH, U.S. National Library of Medicine
					Beijing Mario Biotech Company		
					Hebei Senlang BIotech Company		
					Beijing HuiNengAn Biotech Company		
	EGFRvIII	Phase I	NCT03283631	June 30, 2020	Gary Archer Ph.D.	Terminated	Clinicaltrials.gov, NIH, U.S. National Library of Medicine
					National Cancer Institute (NCI)		
					Duke Cancer Institute		
	MPP2 +	Phase I	NCT04214392	February 6, 2023	City of Hope Medical Center	Recruiting	Clinicaltrials.gov, NIH, U.S. National Library of Medicine
					National Cancer Institute (NCI)		
	B7-H3	Phase I	NCT05241392	December 31, 2024	Beijing Tiantan Hospital	Recruiting	Clinicaltrials.gov, NIH, U.S. National Library of Medicine
	HER	Phase I	NCT01109095	March 7, 2018	Baylor College of Medicine	Completed	Clinicaltrials.gov, NIH, U.S. National Library of Medicine
					The Methodist Hospital Research Institute		
					Center for Cell and Gene Therapy, Baylor College of Medicine		
	GD-2	Phase I	NCT00085930	June 2023	Baylor College of Medicine	Active, not Recruiting	Clinicaltrials.gov, NIH, U.S. National Library of Medicine
					Center for Cell and Gene Therapy, Baylor College of Medicine		
Ovarian Cancer	CD133	Phase I/II	NCT02541370	June 2019	Chinese PLA General Hospital	Completed	Clinicaltrials.gov, NIH, U.S. National Library of Medicine
	Anti-ALPP	Phase I/II	NCT04627740	December 31, 2023	Xinqiao Hospital of Chongqing	Recruiting	Clinicaltrials.gov, NIH, U.S. National Library of Medicine
	Anti-MESO	Phase I	NCT03814447	January 2023	Shanghai 6th People's Hospital	Recruiting	Clinicaltrials.gov, NIH, U.S. National Library of Medicine
					Hrain Biotechnology Co., Ltd		
	CD70	Phase I	NCT05518253	May 30, 2025	Weijia Fang, MD	Recruiting	Clinicaltrials.gov, NIH, U.S. National Library of Medicine
					Chongqing Precision Biotech Co., Ltd		
	TAG72	Phase I	NCT05225363	November 5, 2026	City of Hope Medical Center	Recruiting	Clinicaltrials.gov, NIH, U.S. National Library of Medicine
					National Cancer Institute (NCI)		
	fhB7H3	Phase I/II	NCT05211557	August 31, 2026	The Affiliated Hospital of Xuzhou Medical University	Recruiting	Clinicaltrials.gov, NIH, U.S. National Library of Medicine
					Xuzhou Medical University		
					IIT MediTech (Jiangsu) Co. Ltd		
	αPD1-MSLN	Early Phase I	NCT04503980	June 2022	Shanghai Cell Therapy Group Co., Ltd	Unknown	Clinicaltrials.gov, NIH, U.S. National Library of Medicine
					Shanghai 10th People's Hospital		
	CLDN 18.2	Phase I	NCT05472857	December 2024	Suzhou Immunofoco Biotechnology Co., Ltd	Recruiting	Clinicaltrials.gov, NIH, U.S. National Library of Medicine
					Changhai Hospital		
Lung Cancer	αPD1-MSLN	Early Phase I	NCT04489862	December 2022	Wuhan Union Hospital, China	Recruiting	Clinicaltrials.gov, NIH, U.S. National Library of Medicine
					Shanghai Cell Therapy Group Co., Ltd		
	Anti-MUC1	Phase I/II	NCT03525782	January 31, 2022	The First Affiliated Hospital of Guangdong Pharmaceutical University	Unknown	Clinicaltrials.gov, NIH, U.S. National Library of Medicine
					Guangzhou Anjie Biomedical Technology Co., Ltd		
					University of Technology, Sydney		
	TAA06	Phase I	NCT05190185	December 1, 2023	PersonGen BioTherapeutics (Suzhou) Co., Ltd	Recruiting	Clinicaltrials.gov, NIH, U.S. National Library of Medicine
					Department of Immunology, The Fourth Hospital of Hebei Medical University		
	CEA	Phase I/II	NCT04348643	April 30, 2023	Chongqing Precision Biotech Co., Ltd	Recruiting	Clinicaltrials.gov, NIH, U.S. National Library of Medicine
	P-MUC1C-ALLO1	Phase I	NCT05239143	April 2039	Poseida Therapeutics, Inc.	Recruiting	Clinicaltrials.gov, NIH, U.S. National Library of Medicine
	EGFR/B7H3	Early Phase I	NCT05341492	May 1, 2025	Second Affiliated Hospital of Guangzhou Medical University	Recruiting	Clinicaltrials.gov, NIH, U.S. National Library of Medicine
	CAdVEC and HER2	Phase I	NCT03740256	December 30, 2038	Baylor College of Medicine	Recruiting	Clinicaltrials.gov, NIH, U.S. National Library of Medicine
					The Methodist Hospital Research Institute		
	CEA	Phase I/II	NCT04348643	April 30, 2023	Chongqing Precision Biotech Co., Ltd	Recruiting	Clinicaltrials.gov, NIH, U.S. National Library of Medicine
Prostate Cancer	EpCAM	Phase I/II	NCT03013712	December 2020	First Affiliated Hospital of Chengdu Medical College	Unknown	Clinicaltrials.gov, NIH, U.S. National Library of Medicine
	P-PSMA-101	Phase I	NCT04249947	September 2036	Poseida Therapeutics, Inc.	Recruiting	Clinicaltrials.gov, NIH, U.S. National Library of Medicine
	Anti-PSCA-CAR-4-1BB/TCRzeta-CD19t	Phase I	NCT03873805	December 31, 2023	City of Hope Medical Center	Recruiting	Clinicaltrials.gov, NIH, U.S. National Library of Medicine
					National Cancer Institute (NCI)		
	PD1-PSMA	Phase I	NCT04768608	January 2024	Zhejiang University	Recruiting	Clinicaltrials.gov, NIH, U.S. National Library of Medicine
					Bioray Laboratories		
	BPX-601	Phase I/II	NCT02744287	February 2026	Bellicum Pharmaceuticals	Recruiting	Clinicaltrials.gov, NIH, U.S. National Library of Medicine
Liver Cancer	Anti-CEA	Phase I	NCT05240950	December 25, 2026	Changhai Hospital	Recruiting	Clinicaltrials.gov, NIH, U.S. National Library of Medicine
	IM83	Phase I	NCT05123209	August 30, 2023	Beijing Immunochina Medical Science & Technology Co., Ltd	Recruiting	Clinicaltrials.gov, NIH, U.S. National Library of Medicine
	GPC3	Phase I	NCT05344664	February 1, 2025	Peking University	Recruiting	Clinicaltrials.gov, NIH, U.S. National Library of Medicine
	Anti-CD133	Phase I/II	NCT02541370	June 2019	Chinese PLA General Hospital	Completed	Clinicaltrials.gov, NIH, U.S. National Library of Medicine
	IL15 (AGAR)	Phase I	NCT04377932	February 1, 2040	Baylor College of Medicine	Recruiting	Clinicaltrials.gov, NIH, U.S. National Library of Medicine
					Center for Cell and Gene Therapy, Baylor College of Medicine		
	HerinCAR-PD1	Phase I/II	NCT02862028	July 2018	Shanghai International Medical Center	Unknown	Clinicaltrials.gov, NIH, U.S. National Library of Medicine
	Anti-CEA	Early Phase I	NCT04513431	August 30, 2023	Ruijin Hospital	Not yet Recruiting	Clinicaltrials.gov, NIH, U.S. National Library of Medicine
					HuaDao (Shanghai) Biomedical Co., Ltd		
Pancreatic Cancer	CT041	Phase I/II	NCT04404595	September 1, 2035	CARsgen Therapeutics Co., Ltd	Recruiting	Clinicaltrials.gov, NIH, U.S. National Library of Medicine
	HEC-016	Early Phase I	NCT05277987	March 2025	Shenzhen Fifth People's Hospital	Recruiting	Clinicaltrials.gov, NIH, U.S. National Library of Medicine
	IM96	Early Phase I	NCT05287165	June 30, 2024	Beijing Immunochina Medical Science & Technology Co., Ltd	Recruiting	Clinicaltrials.gov, NIH, U.S. National Library of Medicine
	TAI-meso	Phase I	NCT02706782	September 2018	Shanghai GeneChem Co., Ltd	Unknown	Clinicaltrials.gov, NIH, U.S. National Library of Medicine
	Claudin 18.2	Phase I	NCT05472857	December 2024	Suzhou Immunofoco Biotechnology Co., Ltd	Recruiting	Clinicaltrials.gov, NIH, U.S. National Library of Medicine
					Changhai Hospital		
	KD-496	Early Phase I	NCT05583201	June 1, 2026	Jianming Xu	Recruiting	Clinicaltrials.gov, NIH, U.S. National Library of Medicine
					KAEDI		
	CT041	Phase I/II	NCT04581473	June 30, 2038	CARsgen Therapeutics Co., Ltd	Recruiting	Clinicaltrials.gov, NIH, U.S. National Library of Medicine
					Peking University Cancer Hospital & Institute		
					Fudan University		
	Anti-hCD70	Phase I/II	NCT02830724	January 1, 2028	National Cancer Institute (NCI)	Recruiting	Clinicaltrials.gov, NIH, U.S. National Library of Medicine
Colorectal Cancer	NKG2D	Early Phase I	NCT05248048	October 2022	The Third Affiliated Hospital of Guangzhou Medical University	Recruiting	Clinicaltrials.gov, NIH, U.S. National Library of Medicine
					Hangzhou Cheetah Cell Therapeutics Co., Ltd		
	Anti-CEA	Phase I	NCT05240950	December 25, 2026	Changhai Hospital	Recruiting	Clinicaltrials.gov, NIH, U.S. National Library of Medicine
	αPD1-MSLN	Early Phase I	NCT04503980	June 2022	Shanghai Cell Therapy Group Co., Ltd	Unknown	Clinicaltrials.gov, NIH, U.S. National Library of Medicine
					Shanghai 10th People's Hospital		
	CEA	Phase I/II	NCT04348643	April 30, 2023	Chongqing Precision Biotech Co., Ltd	Recruiting	Clinicaltrials.gov, NIH, U.S. National Library of Medicine
	CEA	Phase I	NCT05415475	September 15, 2024	Chongqing Precision Biotech Co., Ltd	Recruiting	Clinicaltrials.gov, NIH, U.S. National Library of Medicine
	Anti-CD133	Phase I/II	NCT02541370	June 2019	Chinese PLA General Hospital	Completed	Clinicaltrials.gov, NIH, U.S. National Library of Medicine
	Anti-HER2	Phase I/II	NCT02713984	July 2019	Zhi Yang	Withdrawn	Clinicaltrials.gov, NIH, U.S. National Library of Medicine

#### Breast cancer

Of the 19 antigen targets for CAR-T cells in breast cancer, 12 are in ongoing clinical trials [189]. The most common antigen targets include neural cell adhesion molecule L1 (CD171), CEA, fibroblast activation protein (FAP), CA-IX, folate receptor α (FR-α), GD2, MUC-1, EGFR variant III, and VEGF receptor 2 (VEGF-R2) [190–192]. Increased expression of MUC-1 (98.6%) has been reported in invasive breast tumors [193], and MUC28z CAR-T cells derived from single-chain variable fragment (scFv) of TAB004 significantly reduced the growth of triple-negative breast cancer (TNBC) tumors in vitro and in vivo [194]. Additionally, mesothelin-specific CAR-T cells were shown to exhibit increased cytotoxicity towards mesothelin-expressing primary breast cancer cells [195], and human CD3 + T-cells with anti-ERBB2-CAR were found to induce apoptosis in ERBB2-overexpressing breast cancer cells [196], suggesting ERBB2/HER2 as an important target for inducing cytotoxicity in ERBB2-expressing tumors. The incorporation of ligand-based CARs was reported to warrant high affinity to tumor cells and minimize the immunogenicity of chimeric proteins compared to the traditional humanized scFvs [197]. Heregulin-1β (HRG1β), an endogenous ligand for HER3/HER4-based CAR-T cells, suppressed the growth of HER3-positive breast cancer cells in vitro and in vivo [198].

#### Prostate cancer

In prostate cancer, TAAs such as PSMA, prostate stem cell antigen (PSCA), prostate-specific antigen (PSA), and epithelial cellular adhesion molecule (EpCAM) are being investigated [199]. PSMA-based CAR-T cells constitutively expressing inverted chimeric cytokine receptor (ICR) exhibit significant antitumor effects on prostate cancer cells in vitro and in vivo [200]. Furthermore, phase I clinical trial studies have shown the efficacy and safety of using CAR-T cells targeting PSMA in patients with prostate cancer [201–203].

#### Ovarian cancer

Aberrantly expressed glycosylated cell surface proteins are attractive immunotherapy targets, including for CAR-T cells. In ovarian tumors, such proteins include MUC-1, mucin-16 (MUC-16), and tumor-associated glycoprotein 72 antigen (TAG72) [204–206]. PD1-antiMUC16 [207] and TAG72-specific CAR containing a 4-1BB intracellular co-stimulatory signaling domain (TAG72-BBζ) [208] show cytotoxicity against ovarian cancer cells in vitro and in vivo*.* In another study, T-cells modified to express 4H11-28z CAR lysed ovarian cancer cells in vitro and exhibited in vivo antitumor activity in SCID mice bearing orthotopic human MUC-CD + ovarian carcinoma tumors [209]. Another protein, 5T4, an oncofetal TAA highly expressed in ovarian cancer [210], is also an attractive target for CAR-T cell therapy: co-culturing CAR-T cells, engineered from patient-derived T cells to target the 5T4 antigen, with autologous 5T4 + tumor cells often results in increased secretion of pro-inflammatory cytokines such as IFN-γ and IL-2. This phenomenon is part of the CAR-T cells' immune response against the tumor cells, and can be used as a measure of their reactivity against the tumor antigen [211]. In addition, NSG mice treated with 5T4-specific CAR-T cells showed prolonged survival in a dose-dependent manner [211].

#### Glioblastoma

In glioblastoma, EGFR variant III, ERBB2/HER2, and IL-13 receptor α2 (IL13Rα2) are important CAR-T cell therapy targets that have been investigated in many clinical trial studies [212]. Several studies have shown the antitumor efficacy of EGFR variant III-directed CAR-T cells against glioblastoma [213–215]. Intracranial administration of CAR-T cells targeting IL13Rα2, a monomeric high-affinity receptor for IL-13 found to be overexpressed in glioblastomas [216], improves T-cell persistence and antitumor efficacy against glioblastoma [217, 218]. A study has also shown the therapeutic efficacy of third-generation anti-HER2 in combination with PD-1 blockade against glioblastoma cells [219]. A phase I trial study highlighted the safety, efficacy, and clinical benefit obtained by using HER2-specific, CAR-modified virus-specific T-cells to treat progressive glioblastoma [220]. In a recent study, disialoganglioside GD2 antigens were an important therapeutic target for CAR-T cell therapy in glioblastoma [221]. GD2-CAR-T cells exhibited an antitumor effect against GD2 + glioblastoma cells and improved survival in the orthotopic glioblastoma model [221].

#### Colorectal cancer

In colorectal cancer (CRC), several antigen targets for CAR-T cell therapy are being investigated in clinical trials, including CEA, CD133, C-Met, EGFR, HER2, EpCAM, MUC-1, mesothelin, PSMA, guanylate cyclase-C (GCC), and natural killer group 2 member D ligand (NKG2DL) [222]. NKG2D CAR-T cells exhibited cytotoxicity against human CRC cells and suppressed tumor growth in a xenograft model of CRC [223]. In another study, GUCY2C-targeted murine CAR-T cells promoted antigen-dependent T-cell activation in GUCY2C-expressing cancer cells in vitro and provided long-term protection in a syngeneic lung metastasis mouse model [224]. Clinical trial studies have also demonstrated the safety, tumor trafficking, and immunogenicity of TAG-72 CAR-T cells in CRC [225]. A recent study showed the use of Doublecortin-like kinase 1 (DCLK1)-based CAR-T cells as a treatment strategy for eradicating CRC tumor stem cells [226]. The study showed that DCLK1-based CAR-T cells exhibited cytotoxicity against CRC cells and inhibited CRC tumor growth in vivo [226]. A previous clinical trial study also demonstrated the efficacy and tolerability of CEA CAR-T cells in CEA^+^ refractory CRC patients with liver and lung metastasis [227].

#### Lung cancer

Lung cancer has remained one of the most frequently occurring cancers worldwide, with a high mortality rate and poor prognosis, making it an essential target for CAR-T cell therapy. Current antigen targets in clinical trials for CAR-T cell therapy for lung cancer include MUC-1, CEA, HER2, mesothelin, receptor tyrosine kinase-like orphan receptor 1 (ROR1), glypican-3 (GPC3), EGFR, and PD-1 [228]. CAR-T cells targeting EGFR variant III [229], mesothelin [230], Erythropoietin-producing hepatocellular carcinoma A2 (EphA2) [231], Delta-like 3 (DLL3) [232], PSCA- and MUC-1 [233], and PD-L1 [234] have shown significant antitumor effects against lung cancer cells in vitro and in vivo. In another study, ROR1 CAR-T cells have been found to be effective in inducing apoptosis of 3D lung cancer tumors in static culture [235].

#### Hepatocellular carcinoma

Hepatocellular carcinoma (HCC) accounts for the majority of primary liver cancers and represents a significant cause of cancer-related mortality worldwide, although it is not the most common cause [236]. The liver is the most common site for cancer metastasis originating from other organs, such as the pancreas, rectum, colon, breast, and lung [237]. The current HCC antigen targets in clinical trials for CAR-T cell therapy include GPC3, EpCAM, Claudin 18 (CLD18), CD147, EGFR variant III, C-Met, and death receptor 5 (DR5) [238]. Dual-targeted CAR-T cells co-expressing GPC3 and asialoglycoprotein receptor 1 (ASGR1) exhibited cytotoxicity against GPC3^+^ASGR1^−^ and GPC3^+^ASGR1^+^ HCC cells in vitro and caused a significant reduction of GPC3^+^ASGR1^+^ HCC tumor xenografts in vivo [239]. Another study showed GPC3 CAR-T cells co-expressing interleukins IL-15 and IL-21 to exhibit cytotoxicity against HCC cells in vitro and show superior expansion, persistence, and potent antitumor activity against HCC in vivo [240].

#### Pancreatic cancer

Pancreatic cancer is the most common type of pancreatic malignancy, with a poor survival rate and dismal prognosis due to the lack of effective systemic therapies and limited treatment options. The major cause of the limited success of immunotherapy in pancreatic ductal adenocarcinoma (PDAC), the most common histological subtype of pancreatic cancer, is the presence of a highly immunosuppressive TME [241]. CAR-T cell therapy represents an emerging therapeutic option for overcoming these immunosuppressive elements in PDAC. Current pancreatic cancer antigen targets in clinical trials for CAR-T cell therapy include CEA, mesothelin, CD133, CD70, CLD18, HER2, GPC3, PSCA, EGFR variant III, and MUC-1 [242]. CAR-T cells targeting a type I transmembrane protein B7-H3 exhibit antitumor activity against PDAC cells in vitro and controlled tumor growth in a xenograft mouse model using PDAC patient-derived tumors [243]. Anti-Tn-MUC1 CAR-T cells [244] and CXCR2-expressing CAR-T cells [245] have also shown superior antitumor activity against such models. A phase I clinical trial study has demonstrated the safety and feasibility of using HER2 CAR-T cells against advanced pancreatic cancers [246].

In recent years, CAR-T cell therapy in solid tumors has shown limited success because of tumor antigen heterogeneity, immunosuppressive TME, CAR-T cell trafficking and tumor infiltration, and on-target off-tumor effects that restrict the efficacy of CAR-T cell therapy within solid tumors [68]. Overcoming these limitations may enable treatment of solid tumors using CAR-T cell therapy to become as successful as it is in hematological malignancies. Various engineering strategies, such as fine-tuning the affinity of CARs to their cognate antigens [247–249] or engineering CARs that target tumor-associated glycopeptide epitopes [244, 250–252] can improve the targeting-specificity of CAR-T cells. Other strategies include developing CARs capable of Boolean-logic signal integration and suicide systems that can eliminate engineered T-cells [253]. The capability of CAR-T cells could also be enhanced by using a split, universal, and programmable (SUPRA) CAR system that enables switching targets without re-engineering T-cells, fine-tunes T-cell activation, and enables the integration of signals from multiple antigens [254]. Overall, a multi-faceted approach involving novel T-cell engineering approaches and gene-editing techniques will allow us to make incremental changes to navigate through the barriers presented by the complex TME of solid tumors to deliver effective CAR-T cell therapy.

## CAR-T cell therapy limitations and their mitigation strategies

**Fig. 2:**
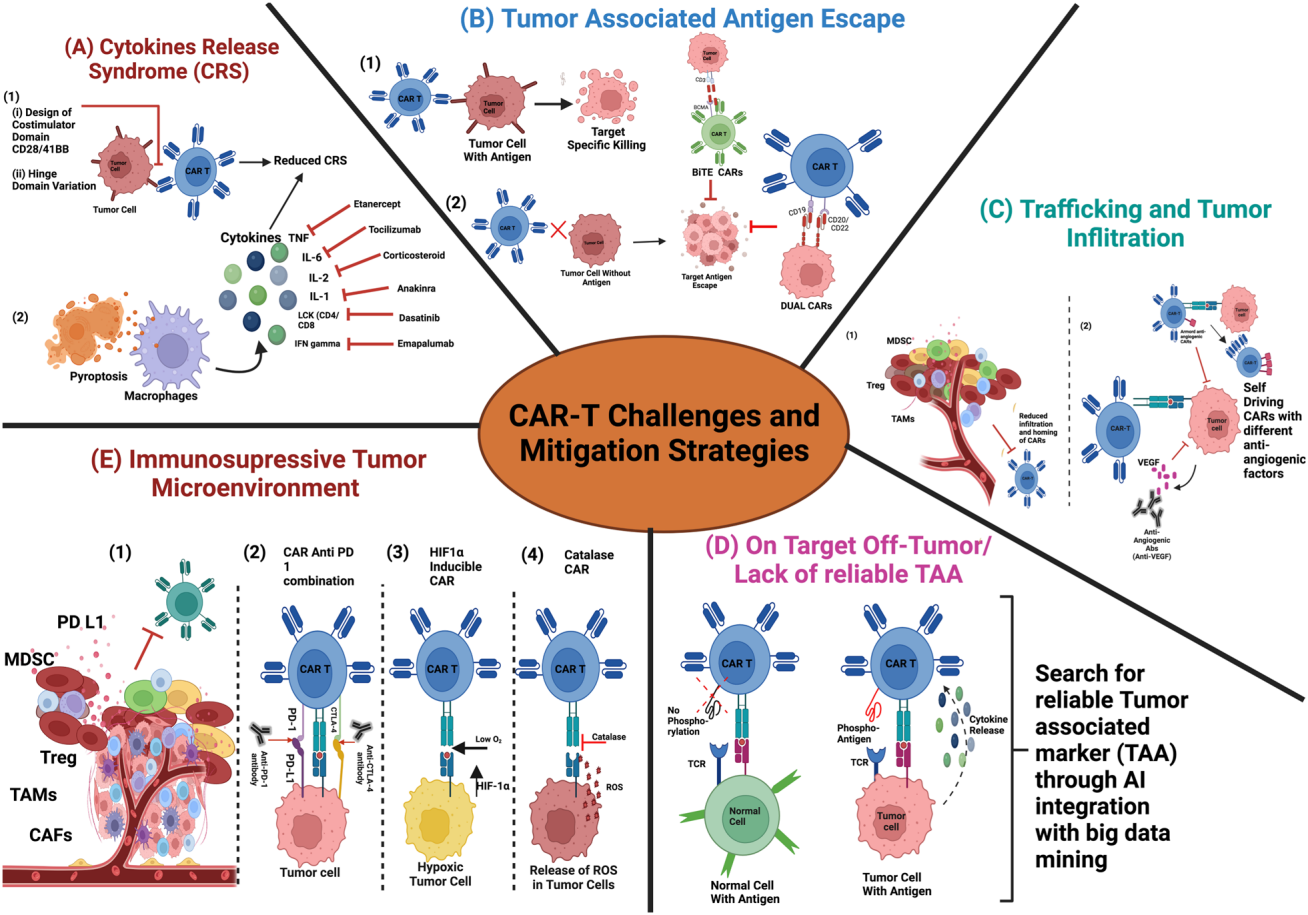
CAR-T cell therapy challenges and their mitigation strategies. **A** *Cytokine Release Syndrome (CRS)* (1) Choice of costimulatory domain CD28 or 41BB as well as the length of the hinge domain influence CRS (2) Cytokines released by macrophages and Inflammatory cytokines and immunostimulatory alarmins released during pyroptosis can be mitigated by using specific drugs for each cytokine (e.g., Etanecerpt, Tocilizumab, Corticosteroids, Dasatinib, Emapalumab) **B** *Tumor-associated antigen escape* (1) CAR-T cell-mediated killing of target cell if the target antigen is present on the surface (2) Tumor antigen escape in the absence of surface antigen of the CAR-T cell and potential strategies to abet it by using DUAL CARs and BiTE CARs. **C** Trafficking and tumor infiltration (1) Schematic diagram to demonstrate reduced homing of CAR-T cells to tumor microenvironments due to the presence of different cellular components (2) Improving homing of CAR-T cells to TME by using armored anti-angiogenic CARs as well as self-driving CARs, which express multiple anti-angiogenic factors. **D** *On-target Off-tumor/Lack of reliable TAAs.* Schematic diagram to demonstrate targeting of the normal cell by CAR-T cells if the antigen is expressed on normal cells, which can be mitigated by a selection of reliable tumor-associated antigen by integration of artificial intelligence with big data mining. **E** *Immunosuppressive tumor microenvironment* (1) Diagram to illustrate suppressive tumor microenvironment comprising different cellular components including low oxygen, cancer-associated fibroblast, high ROS and other components that diminish proliferation of CAR-T cells (2) CAR-T expressing anti-checkpoint inhibitors to promote the growth of T cells in tumor microenvironments (3) HIF1α-inducible CARs, which get activated in hypoxic tumor microenvironment. HIF1α to promote T cell growth (4) Catalase-expressing CAR to scavenge reactive oxygen species in tumors to promote T cell growth. CARs, Chimeric antigen receptors; BiTE, bispecific T-cell engagers; TAAs, tumor-associated antigens; TME, tumor microenvironment; ROS, reactive oxygen species; HIF1α, hypoxia inducible factor 1 alpha

CAR-T cell therapy has immense potential, which is evident by the approval of various forms of CAR-T cell therapies for the treatment of cancer and by the stable remission seen in patients after treatment by CARs directed against the different antigens. CAR-based therapies are projected to hold tremendous promise in the near future; however, many limitations of CAR-T therapy must be addressed before the CARs become universally acceptable, especially in solid cancers (Fig. 2). In the upcoming section, we will discuss the unique issues faced by CAR-T cell therapy in hematological and solid cancers and the efforts to address these issues.

### Cytokine release syndrome

Cytokine release syndrome (CRS) is one of the most common toxicities associated with the infusion of CAR-T cells. The nature of CRS in response to CAR-T infusion and its abetment is an active research area. CRS is thought to occur when CAR T-cells, upon recognizing their target antigen on cancer cells, become activated and start to proliferate and release inflammatory cytokines. These cytokines, in turn, can activate other immune cells, including monocytes and macrophages. Monocytes and macrophages play a crucial role in the pathogenesis of CRS. These cells express high levels of the Fc receptor, which can bind to the Fc portion of the monoclonal antibodies used in CAR T-cell therapy. Upon activation, monocytes and macrophages can produce large amounts of inflammatory cytokines such as IL-6 and IL-1, amplifying the inflammatory response and contributing to the symptoms of CRS [255–257]. Major symptoms of CRS in patients treated with CAR-T cells are high fever, muscle and joint pain, low blood pressure, nausea, fatigue, headache, and skin rashes [258]. Toxicities due to CRS are generally reversible with supportive care, but it is widely thought that the magnitude of CRS varies from patient to patient, as well as by the type of CAR being used.

Many strategies have been used to mitigate CRS, including the blockade of IL-6 receptors and corticosteroids (Fig. 2a). Some of the FDA-approved monoclonal antibodies for the treatment of CRS after CAR-T cell therapy are Tocilizumab [55, 259], Siltuximab [260], and Sarilumab [261]. Tocilizumab and Siltuximab bind and block the IL-6 receptor. Both drugs are widely used as they reverse the CRS symptoms in most patients in a very short time. When CAR-T cell therapy refracts to anti-IL-6 therapy, the use of corticosteroids is considered because it suppresses T-cell function [262]. In ZUMA 1 trial, Tocilizumab reduced the incidence of CRS from 13 to 5% [263]. A simultaneous report that coadministration of Tocilizumab and steroids reduced incidence of CRS without affecting the clinical outcome indicate that this drug combination does not interfere with the efficacy of CAR-T cells [264]. The design of CAR-T also affects the CRS in patients with factors such as the nature of the co-stimulatory domain and intrinsic and extrinsic cellular features influencing the outcome (Fig. 2a). CD19 CAR-T cell efficacy depends on the antigen density of the target cell, with CARs having CD28 as co-stimulatory domain showing better efficacy than 4-1BB construct with a lower risk of CRS [265]. Additionally, the length of endodomain can affect the CAR functionality as well as CRS, with CARs having shorter intracellular domains and CD28 as a costimulatory domain having reduced interaction with CD3ζ, leading to less cellular activation and cytokine production [266]. Conversely, CD19 CARs with longer transmembrane domains showed less CRS than did CARs with shorter domains in a clinical trial [267]. The nature of the transmembrane moiety also influences CRS, with CD19 CARs having a transmembrane moiety containing CD8α producing fewer cytokines and lower levels of activation-induced cell death than CD28 CARs with comparable efficacy to eliminate tumors in preclinical models [62]. A phase 1 clinical trial with fully humanized anti-CD19 CAR with CD8α transmembrane showed significantly reduced level of cytokines as compared to construct used in ZUMA 1 trial [268].

Selective apoptosis has also been explored as a possible strategy to mitigate CRS by using an inducible form of caspase 9 (iCasp9) that is activated upon exposure to small-molecule AP19013 by dimerization, leading to rapid depletion of infused cells [269, 270]. This approach leads to accelerated depletion of CAR-T cells, resulting in significant loss of anti-tumor activity, so it should be used only in patients experiencing high-grade CRS that could lead to life-threatening complications.

CARs with selective on/off switches have also been explored to regulate CRS. A self-cleaving site, which is a substrate for protease paired to a degron (degradation signal) that induces proteolysis of the CAR protein, has been explored; self-cleavage and proteolysis of degron will turn this CAR to the ON state, leading to the expression of CAR on the cellular surface; administering the protease inhibitor asunaprevir switches this CAR to the OFF state because the active degron moiety induces degradation of CAR degron protein (Fig. 2a) [271]. Tyrosine kinase inhibitors like Dasatinib temporarily disrupt cellular signaling downstream of CD3ζ by inhibiting phosphorylation of lymphocyte-specific protein tyrosine kinase (LCK), so it can be used to fine-tune CAR function and to modulate CRS in patients [272]. Ibrutinib inhibits Bruton’s tyrosine kinase, which is highly expressed in B cell malignancies; this inhibitor increases anti-tumor activity in synergism with CD19 CAR-T cells, simultaneously lowering the severity of CRS in patients with CLL [273].

Immune Effector Cell-Associated Neurotoxicity Syndrome (ICANS) is a significant and adverse reaction often associated with CAR-T cell therapy [274]. ICANS tends to manifest within days to weeks following the infusion of the CAR-T cells. It is marked by an array of neurological symptoms that can vary in severity, from being merely inconvenient to posing serious, life-threatening conditions. Patients might exhibit symptoms such as confusion, restlessness, delirium, and in severe instances, seizures. Extremely grave cases may also present cerebral edema—a dangerous swelling of the brain—and encephalopathy, a general term for diseases that alter brain function or structure. While the precise etiology of ICANS remains unclear, it's postulated that the syndrome results from an excessive release of cytokines and other pro-inflammatory molecules by the genetically modified CAR-T cells. This leads to a potent immune response within the brain. However, the severity of ICANS can differ greatly between patients, making its prediction and management challenging [275]. For those undergoing CAR-T cell therapy, meticulous monitoring is critical to manage the potential risk of ICANS. Regular neurological evaluations are vital, as is paying careful attention to any shifts in the patient's mental status. If symptoms of ICANS are detected, treatment protocols may involve general supportive care, encompassing the use of antiseizure medications or anti-inflammatory drugs to control and reduce neurological symptoms. For severe cases that involve life-threatening brain swelling or changes in brain function, admission to an intensive care unit might be required. Here, patients can be closely observed, and more potent interventions, such as corticosteroids and other immunosuppressive therapies, can be employed. To minimize the risk of ICANS, it is crucial to thoroughly assess patients prior to CAR-T cell therapy to determine their suitability for this treatment. Risk factors that could contribute to the development of ICANS include high disease burden and a high dosage of CAR-T cells. There's a continuous effort in the scientific community to understand the mechanisms underlying ICANS better, with research focused on developing strategies to predict, prevent, and manage this potential severe side effect of CAR-T cell therapy [276, 277]. This research is pivotal in making CAR-T cell therapy safer and more efficient, thereby benefiting more patients in the long run.

### Tumor antigen escape

One of the most challenging limitations of CAR-T cell therapy is the development of tumor resistance to single antigen-targeting CAR-T cells after the initial high response rate [68, 278]. Initial data regarding tumor antigen escape have emerged predominantly from evaluating a patient who received anti-CD19 CARs with the complete or partial loss of CD19 expression leading to resistance to anti-CD19 CAR [279]. Tragocytosis, which is the transfer of antigen from the tumor to the CAR-T cell, has also been reported in a mouse model of leukemia. Reversible antigen loss occurred via transfer of target antigen to T cells, resulting in reduced target density on cancerous cells and thus compromising T cell activity by T cell anergy [280]. This mechanism was reported in both CD28- and 41-BB-based CARs [259].

The use of bispecific CARs has been the primary approach [281, 282] to counter antigen escape, with CARs being developed that can recognize both CD19 and CD20 or CD19 and CD22; these CARs showed better activity than anti-CD19 CAR-T cells did (Fig. 2b) [283, 284]. However, how effective these approaches are remains to be seen. Pan et al. demonstrated that in pediatric B-cell acute lymphoblastic leukemia (B-ALL), sequential infusion of two different CARs targeting CD19 followed by one targeting CD22 could be a viable strategy to increase the efficacy and safety of CAR-T cell therapy [285]. Another important strategy that has emerged recently has been the development of Bi-specific T cell engagers (BiTEs) [286] (Fig. 2b). Blinatumomab, a CD19-specific BiTE, was approved by the FDA to treat B cell precursor ALL (B-ALL) [287]. Much like CAR-T cells, BiTEs (recombinant proteins that simultaneously bind 2 different antigens) facilitate the T-cell-mediated killing of malignant cells by redirecting autologous T lymphocytes to cell-surface antigens on cancer cells The engagement of T cells by BiTEs and CARs is indeed independent of the specificity of the endogenous T-cell receptor (TCR). This is because both BiTE and CAR designs bypass the traditional TCR recognition of antigens presented by the major histocompatibility complex (MHC) on the tumor cells. Instead, they directly recognize the antigen on the cancer cells, which enables the activation of T cells even in the absence of MHC antigen presentation, overcoming a common method by which tumors evade immune surveillance. To clarify, the BiTE platform employs one ScFv for the specific recognition of a tumor-associated antigen and another ScFv to engage the CD3 component of the TCR complex on T cells. The dual specificity of BiTE molecules effectively bridges T cells and cancer cells, facilitating cytotoxic T-cell responses against the tumor [286, 288]. Combining both platforms can be a viable strategy to improve the efficacy of CAR-T cell therapy. CARs can be engineered to secrete BiTEs, which can further enhance the anti-tumor response of CAR-T cells. This strategy has been explored to overcome challenges posed by solid tumors and to overcome antigen escape [289]. In this study, Choi et al. developed a bicistronic CAR against the glioblastoma-specific tumor antigen EGFRvIII and a BiTE against EGFR, which is an antigen frequently overexpressed in glioblastoma. CART. BiTE secreted BiTEs specific to EGFR and recruited un-transduced bystander T cells against wild-type EGFR, thus amplifying the immune response to achieve a better therapeutic outcome.

Tumor heterogeneity is often associated with solid cancer, which further leads to antigen heterogeneity. Furthermore, much like hematological malignancies, there is always a possibility of antigen loss as well as an escape. For example, in a Phase 1 study of an EGFRvIII-specific CAR in GBM, a single dose of the CAR-T cells led to downregulation of the EGFR/EGFRvIII receptor [214], whereas IL13Ra2-specific CAR-T cells co-expressing IL-15 were potent against glioma but led to the rise of tumors with IL13Ra2 downregulation [290]. Much like hematological malignancies, bispecific or tandem CARs have been developed to address the issue of antigen loss or tumor heterogeneity. Bispecific CAR has been developed against HER2 and MUC1 as well as against HER2 and IL13Ra2; both are in the preclinical development phase [291]. Whether these CARs are successful enough to progress to phase 3 trials remains to be seen.

### Trafficking and tumor infiltration

The biggest challenge for using CAR-T cells in solid cancers is getting them to the target site. This is not an issue in hematological cancers as circulating T cells are very near their target. In solid tumors, CAR-T cell therapy is limited due to the inability of these cells to traffic and infiltrate the tumor due to the immunosuppressive TME and physical tumor barriers such as stroma that limit the diffusion and mobility of CAR T-cells (Fig. 2c). A T cell must cross many barriers before it encounters the tumor cells and their associated antigen. Generally, the trafficking of T cells to a particular site is controlled by a chemotactic gradient, so if the T cells are not expressing the appropriate chemokine receptor, then the T cell cannot reach their targets [292, 293]. Moreover, T cells encounter many physical barriers and abnormal vasculature at the tumor site, such as high endothelial venules, which facilitate T cell entry but are distorted in tumors [294]. The potential solution for overcoming abnormal vasculature can be the administration of anti-angiogenic therapy targeting angiogenic factors such as VEGF, which normalizes vasculature with CAR T cell therapy (Fig. 2c) [295]. To address the issue of tumor trafficking of T cells, one of the strategies has been to add the chemokine receptor expression on CAR T-cells that match and respond to the chemokines derived from the target tumor [245]. The physical barrier, tumor stroma, that prevents the penetration of CAR T-cells, is mainly composed of an extracellular matrix of heparin sulfate proteoglycan (HSPG). HSPG does not let CAR-T cells pass into the tumor [296]. Engineered CAR-T cells expressing heparinase can degrade HSPG and lead to enhanced tumor infiltration and elimination [297]. In animal models, fibroblast activation protein (FAP)-targeted CAR-T cell therapy showed a reduction in tumor fibroblasts by increasing the cytotoxic function [298]. Researchers have developed a chemokine CXCL11/mesothelin CAR, which increases the intratumoral level of CXCL11 to aid the migration of CAR T cells to the target site [299]. Furthermore, another armored mesothelin CAR-T cell expresses both IL-7 and CCL19 and has shown promising results in a murine model [300].

A good strategy to promote the trafficking of T cells is the administration of T cells to the site of tumor itself. In patients with metastatic breast cancer, intratumoral administration of mRNA c-Met CAR T cells result in tumor regression and macrophage recruitment [301]. In the murine model, considerable success has been demonstrated using this strategy. Intratumoral delivery of a HER2-BBz CAR T cell led to the regression of medulloblastomas in NSG mice at a dose significantly lower than that for intravenous delivery of this CAR [302]. The effectiveness of this approach was shown in a study in which intracavitary administration of pan-ErbB/IL-4 CAR T cells targeting patient-derived MPM xenografts in severe combined immunodeficient (SCID) mice showed significant tumor regression and cure in all mice [303]. In another study, intracranial and intratumoral administration of HER2-specific CAR T cells showed better antitumor activity than intravenous delivery, along with complete tumor regression and 100% survival following tumor rechallenge [304].

### On-target off-tumor toxicity

One of the toxicities observed in CAR-T cell therapy is that tumor antigens are also expressed on normal tissues at variable levels, leading to attack against normal tissues and, thus, toxicity [305] (Fig. 2d). B cell maturation antigen (BCMA) is a widely used target for immunotherapy. These antigens are highly expressed in mature B cells, including plasma cells, and few are present in other cell lineages [149, 306, 307]. CAR-T cell therapy can lead to secondary hypogammaglobulinemia, as BCMA is also expressed in healthy plasma cells. Similarly, CD19-positive B cells are affected by anti-CD19 CAR T-cell therapy, which can cause B cell aplasia [308]. CD38 antigen, also expressed in the gastrointestinal tract, cerebellar Purkinje cells, or even T cells, is another immune target for plasma cells [309–311]. On-target off-tumor toxicity should be considered when targeting CD38 with CAR T-cells. Targeting tumor-restricted post-translational modifications overexpressed by solid tumors such as truncated O-glycans such as Tn (GalNAcal-O-Ser/Thr) and sialyl Tn (NeuAca2-6-GalNAcal-O-Ser/Thr) can help overcome the on-target off-tumor toxicity effects [312]. CAR-T cell local administration to the disease site is another approach that might limit on-target-off tumor toxicity [68].

### Lack of reliable tumor-associated antigens in solid cancers

The TME in solid cancer is highly heterogeneous unlike that in hematological cancers such as ALL, CLL, and MM, where there is uniform expression of antigens. However, in almost all solid cancers, TAA are expressed not only on cancer cells but also on normal tissue. Examples of this phenomenon include the common targets EGFR, Mesothelin, MUC1, and PSMA [313–315]. The on-target off-target problem in solid cancers has led to catastrophic outcome in some clinical trials: in a metastatic colon cancer trial, CAR-T cells targeting HER2 antigen led to mortality of the patient 5 days after infusion [316] (Fig. 2d) because the low expression of HER2 in lung epithelium led to the collapse of both lungs. In a neuroblastoma study, high-affinity CAR targeting GD2 led to fatal encephalitis as this antigen is expressed at a low level in brain tissues. These studies point out two critical issues with CARs in solid cancer. First, the antigen selection in solid cancer should be made only after extensive preclinical studies to ascertain the expression in normal tissues. Second, higher-affinity binding CARs are not necessarily better in terms of efficacy and safety. This principle is illustrated by examining the case of ICAM-1, which is expressed in many solid tumors and normal tissues. The CAR that had affinity in micromolar concentration was more effective than was the CAR that had affinity in nanomolar concentration, showing less anergy and increased proliferation [317]. This on-target off-tumor effect can cause damage to normal tissues and induce side effects ranging from manageable (like skin rashes or fevers) to severe and even life-threatening, such as neurotoxicity or CRS [171]. To mitigate this issue, researchers are developing strategies like dual antigen targeting (requiring the presence of two antigens instead of one to activate the T cells), inducible suicide genes (that can be activated to kill off the T cells if severe toxicity occurs), and antigen-binding domains that preferentially bind to antigens at high densities (like those found on tumor cells) [173]. It's also important to note that the selection of suitable tumor-specific or tumor-associated antigens with limited expression on vital normal tissues is key for the safety and effectiveness of CAR T cell and BiTE therapies.

In the age of-omics, many strategies have been developed, including DNA/RNA sequencing of exome to identify a mutation in tumors. Whole-genome sequencing found antigen-specific TILs in five patients [318]. Balachandran et al. used whole-exome sequencing and in silico neoantigen prediction in pancreatic ductal adenocarcinoma to identify new TAA and found MUC16 (CA125). Loss of these MUC16 neoantigen clones was seen on metastasis, suggesting neoantigen immunoediting to be a phenomenon in patients with PDAC [319].

## Overcoming the immunosuppressive tumor microenvironment

In the TME, various tumor-infiltrating cells, such as myeloid-derived suppressor cells, tumor-associated macrophages, and regulatory T cells, contribute to immunosuppression [320], and the tumor microenvironment is generally considered hostile to T cells. These tumor-infiltrating cells contribute to the production of tumor-facilitating growth factors, cytokines, and chemokines. In the TME, T cell proliferation is inhibited by binding with inhibitory ligands (e.g., programmed cell death ligand 1 [PD-L1] and Galectin-9] and by suppressive myeloid-derived suppressor cells, cancer-associated fibroblasts and other tumor-associated cells, which secrete factors such as VEGF and TGFβ that contribute to an abnormal TME characterized by inflammation and hypoxia (Fig. 2e) [321].

Immune checkpoint proteins such as CTLA-4 or PD-1 contribute to declines in anti-tumor immunity. No response or weak response of CAR-T cells is due to poor T cell expansion and limited T cell persistence in this hostile tumor microenvironment. The development of T-cell exhaustion is provoked by co-inhibitory pathways [322]. To overcome this, combinational immunotherapy with CAR T-cells and checkpoint blockade is a rational approach because it provides two main elements for a strong immune response: better tumor penetration by CAR T-cells and sustained T-cell persistence by PD-1/PD-L1 blockade [323, 324] (Fig. 2e). Immune checkpoint inhibitors have dramatically changed the landscape of cancer treatment by effectively harnessing the immune system to attack cancer cells [325]. These are generally antibodies designed to block inhibitory checkpoint proteins on immune cells, particularly T cells, or on tumor cells, thus "releasing the brakes" on the immune response against cancer cells [326]. Some prominent checkpoint inhibitors include PD-1, PD-L1, and CTLA-4. PD-1 inhibitors target the programmed cell death protein 1 (PD-1) receptor on T cells to prevent their interaction with PD-L1 and PD-L2 on tumor cells, which can inhibit the T cell response [327]. Examples include nivolumab (Opdivo) and pembrolizumab (Keytruda). PD-L1 inhibitors directly target PD-L1 on tumor cells to prevent it from binding to PD-1 and inhibiting T cells. Examples include atezolizumab (Tecentriq), durvalumab (Imfinzi), and avelumab (Bavencio). CTLA-4 inhibitors are inhibitory receptors on T cells. CTLA-4 inhibitors help to amplify the T cell response against cancer. Ipilimumab (Yervoy) is a well-known CTLA-4 inhibitor. Several other potential immune checkpoints are currently under investigation. These include LAG-3, TIM-3, TIGIT, and VISTA, among others. Moreover, the combined use of immune checkpoint inhibitors and other immunotherapies, such as CAR T-cell therapy or BiTEs, is an active area of research [328]. The rationale behind this combination is that while CAR T cells and BiTEs can effectively target and kill cancer cells, the immunosuppressive tumor microenvironment can limit their efficacy. Therefore, by combining these therapies with immune checkpoint inhibitors, it may be possible to enhance the anti-tumor activity of the engineered T cells or engaged T cells. The combinations are currently under investigation in many preclinical models and clinical trials. For example, the combination of pembrolizumab (PD-1 inhibitor) and axicabtagene ciloleucel (a CAR T-cell therapy) is being explored in clinical trials for refractory large B-cell lymphoma.

The first immune checkpoint inhibitor to show efficacy in the clinic is ipilimumab, a monoclonal antibody (mAb) targeting the inhibitory receptor cytolytic T lymphocyte antigen 4 (CTLA-4) [11]. The treatment has led to profound benefits in patients with melanoma. Research into blocking CTLA-4, an immune checkpoint receptor on T cells, paved the way for the discovery of other immune checkpoint receptors and their ligands [11]. These include Programmed Death-1 (PD-1), another inhibitory receptor on T cells, and its ligands, PD-L1 and PD-L2, which can be expressed by tumor cells and other cells in the tumor microenvironment. Other potential immune checkpoint targets include lymphocyte activation gene 3 (LAG-3), T-cell immunoglobulin and ITIM domain (TIGIT), and T-cell immunoglobulin and mucin-domain containing-3 (TIM-3), all of which are expressed on T cells [329, 330]. The identification of these inhibitory pathways has spurred the development of a variety of immune checkpoint inhibitors to block these interactions and enhance anti-tumor immune responses [331]. Monoclonal Ab therapy (atezolizumab, pembrolizumab, nivolumab) directed against the checkpoints mentioned above and inhibitory receptors have shown promise in preclinical animal studies and clinical trials for melanoma, lung cancer, head and neck carcinoma, and other cancer types [332].

Anti-PD-L1 (atezolizumab) and anti-PD-1 (pembrolizumab) are used to treat metastatic NSCLC and are being studied for use in other solid tumors and as promising candidates for combination CAR-T cell therapy. The FDA recently approved anti-PD-1 monoclonal antibody for first-line use in combination with chemotherapy in lung cancer [333]. The first large-scale trial with anti-PD-1 mAb therapy using nivolumab (a human IgG4 monoclonal antibody) demonstrated a 2-year durable response rate in patients with melanoma, and the outcome was strikingly better than that of conventional chemotherapy [21, 334]. Nivolumab treatment also caused less toxicity than conventional therapy [335]. These promising results led to the approval of nivolumab as the first line of treatment for melanoma in Japan and the United States [336]. Clinical trials for treating patients with non-small-cell lung cancer, renal cell carcinoma, and B and T Hodgkin’s lymphoma by using anti-PD-1 mAb rather than conventional therapy showed enhanced clinical benefit, with improvements in overall and progression-free survival [20]. In addition to blocking PD-1, blocking the ligand PD-L1 using anti-PD-L1 mAb was also effective against various cancers, including bladder, head and neck, and renal cell cancer [337, 338].

Ipilimumab lengthened the survival of metastatic mesothelioma patients in a phase III study in 2010. It has shown promising results in mouse mesothelioma models and in many other preclinical studies [339, 340]. To treat metastatic melanoma, ipilimumab has been used in combination with a VEGF inhibitor and is in a phase 1 trial. In this study, anti-VEGF antibodies combined with anti-CTLA-4 therapy resulted in an increase in anti-tumor response resistant to the immunosuppressive effects of the ligand galectin-1 [341]. CRISPR/Cas9 is also being studied for both PD-1 and LAG-3 in CD19-BBζ CAR T cells to knock out the gene for the IR (Inhibitory Receptor) itself. In both cases, tumors were eradicated in mouse xenograft models using the IR knockout CAR-T cells [342, 343].

Activation of T cells is associated with a change in respiration pattern, with T cells switching from oxidative phosphorylation to glycolysis; however, in TME, glucose is depleted, so glycolytic T cells are deprived of nutrients. Furthermore, glucose Glut 1 receptor is downregulated in T cells, resulting in reduced viability of T cells [344]. In addition to nutrient depletion, the hypoxic core of solid cancer also poses a challenge for CAR-T cell activity by limiting oxidative phosphorylation. It provides a significant challenge for memory T cells, whose metabolism relies predominantly on oxygen [345]. CARs’ designs have been modulated to address these issues. Reports indicate that the coreceptor signaling domain influences the metabolism of CAR T cells, and incorporation of the 4-1BB domain stimulated the growth of CD8^+^ central memory T cells, which had elevated respiratory capacity, fatty acid oxidation, and enhanced mitochondrial biogenesis. In contrast, CAR-T cells with CD28 costimulatory domain gave rise to memory cells with an enhanced glycolytic signature [346], indicating that the costimulatory domain characteristic must be considered while designing CARs for hypoxic conditions.

In addition to hypoxia, CARs are also exposed to increases in reactive oxygen species (ROS) in the tumor microenvironment, affecting the viability of CARs. To address this issue, CARs co-expressing catalase enzyme [347], which catalyzes the conversion of hydrogen peroxide to water, have been developed. This CAR-CAT bi-cistronic CAR showed reduced ROS accumulation and robust antitumor activity even in the presence of high ROS. Furthermore, CAR-CAT T cells provided substantial bystander protection of non-transfected immune effector cells (Fig. 2e). In another study, Alexandre et al. developed a CAR in which they fused an oxygen-sensitive subdomain of HIF1α to a CAR domain, thereby generating CAR T-cells that are sensitive to hypoxia’s presence in the tumor core, which stimulates the growth and proliferation of these CAR T cells [348] (Fig. 2e). These studies provided evidence that tailoring CARs to different TMEs is a rational approach to improving CAR’s efficacy.

## Single-cell sequencing for CAR-T cell therapy

Approximately 30–60% of patients receiving CAR-T therapy experience cancer recurrence [349, 350]. Many clinical trials on CAR-T cell therapies have revealed various adverse events and low treatment response rates, as identified in a recent study on 671 registered clinical trials [351]. Conventional CAR-T cell discovery uses in vitro assays and in vivo models to assess the functioning of engineered CAR-T cells by analyzing the expression of cell surface markers, cytokine secretion, and the killing of tumor cells. However, these assays cannot encapsulate the complete picture of T cell immunosurveillance effectors. A comprehensive understanding of cancer cells and TME before, during, and after treatment is needed for a better therapeutic outcome and to overcome therapeutic resistance. The emergence of single-cell RNA sequencing (scRNA-seq) has become a powerful approach to deconvolute heterogeneous cancer cell populations (a complicated network of proliferating malignant cells, immune infiltrates, and tumor stroma) by providing a greater resolution of transcriptome and gene expression patterns [352–358]. Thus, scRNA-seq is a valuable technology for deciphering CAR-T cell therapies and correlating genotypic markers with the clinical outcome.

Similar to unmodified T cells, CAR-T cells exhibit significant heterogeneity that can be intrinsic and produced by CAR design. The CAR-T cell heterogeneity affects efficacy and treatment safety. To understand this heterogeneity among T cell subtypes, single-cell resolution plays a pivotal role by providing the expression profile of each individual cell in the TME and each cell’s association with therapy outcome [359–361]. Although bulk RNA-seq has revealed different genes associated with T cell exhaustion, scRNA-seq revealed additional markers such as Thymocyte selection-associated high mobility group box (TOX), CXCL13, TIGIT, TIM-3, and LAG-3 demonstrating novel ways to overcome exhaustion [362–367].

Efficacy and safety performance of CAR-T cell therapy can be demonstrated during or in the follow-up of adoptive cell transfer (ACT) by using scRNA-seq to analyze the CAR-T cell product. The outcome of the therapy can be monitored by studying high-resolution gene expression, tumor heterogeneity, and cell phylogenies [351, 368]. Following infusion into a patient, CAR-T cells are exposed to a dynamic TME, and how this affects the behavior of CAR-T cells through time is not well studied. A recent study used scRNA-seq along with TCR repertoire sequencing to evaluate the CAR-T cell behavior after infusion [369]. Differential expression of various genes, including those associated with T cell activation (CD69 and CD25), cytokine and chemokine signaling (CCL3, CCL4, and IL2), exhaustion (PD-1, LAG-3, and TIM-3), apoptosis (BCL2 and MCL1), and proliferation (MKI67 and PCNA) was observed at different time points of the treatment, and a profound association with outcome was observed [370, 371]. This paves the way for identifying cell clusters highly enriched in clones with cell proliferative properties. Temporal scRNA-seq data of a few thousand CAR-T cells is highly valuable in monitoring patient progression by identifying predictive biomarkers of clinical response, which can then be used to design personalized therapies [369, 372, 373].

## Use of artificial intelligence to optimize CAR-T cell therapy

The limitations associated with CAR-T cell therapy, including side-effect toxicities, high cost, time duration, and implementation of the latest technology, can be overcome by using artificial intelligence (AI). Further, predicting the efficacy of different immune products, their pathological response, and identifying the optimal products is challenging by using the present, time-consuming, and labor-intensive conventional tools [374]. Enormous clinical and multi-dimensional data on different cancers processed using AI-driven algorithms have transformed cancer treatments with significant improvement in patient outcomes. Using AI to utilize-omics data before and after CAR-T therapy could overcome the limitations and make this approach highly precise. The research in the field of CAR-T cell therapy utilizing AI-based deep learning approaches is sparse, with few studies available [374–376]. AI holds great promise in optimizing CAR-T cell therapy. AI can be employed to analyze large datasets from techniques such as scRNA-seq and TCR-seq, identifying key genetic signatures associated with treatment response [377]. Machine learning algorithms can predict patient response to therapy based on individual characteristics, aiding in the development of personalized treatment strategies [377]. Furthermore, AI can assist in the design of new CAR constructs and the prediction of their efficacy. Spatial transcriptomics, while not yet extensively applied to CAR-T cell research, offers a novel perspective on understanding how CAR-T cells interact with the tumor microenvironment by linking gene expression data with spatial location within the tumor [378]. As this technology advances, its integration with AI could lead to an even deeper understanding of CAR-T cell behavior and potentially reveal strategies to enhance the effectiveness of CAR-T cell therapies.

**Fig. 3:**
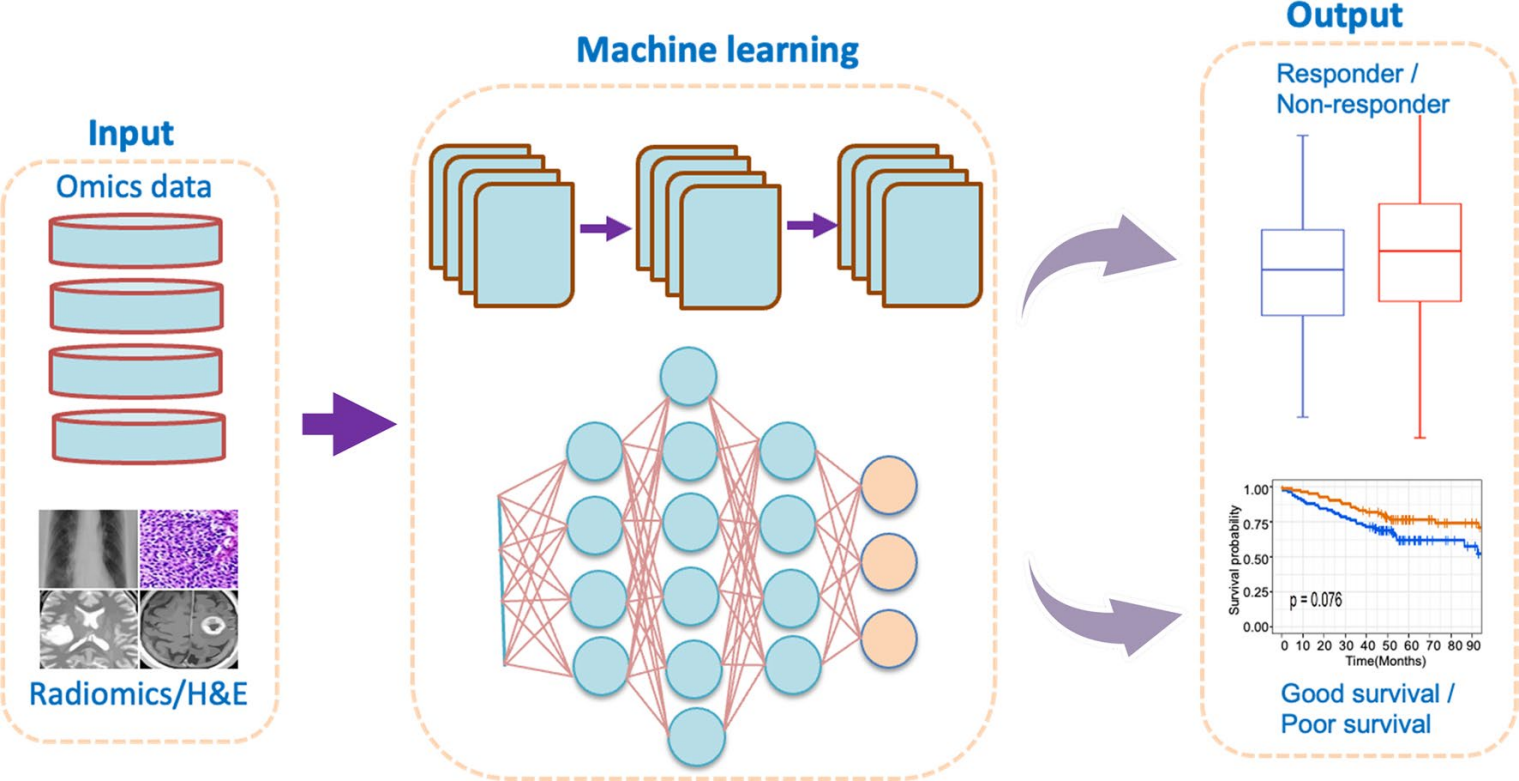
Artificial intelligence-based machine learning model for predicting CAR-T cell therapy outcome. The model can be trained using different-omics and medical imaging datasets as input. The input data are processed by the deep learning algorithm, and results are passed to the output for classification

Several individual biomarkers have been associated with CAR-T therapy response, including lactate dehydrogenase, C-reactive protein, and platelet number [379]. AI-based prognostic tools can be used to identify biomarkers or signatures predicting CAR-T therapy outcomes. Multiple dataset biomarkers can be used, including tumor mutation burden, signaling pathways, types of tumor cells from TME, and gene expression, to build these sophisticated AI models for predicting CAR-T therapy response, disease progression, and survival. Radiomics such as computed tomography, positron emission tomography, and magnetic resonance imaging can predict CAR-T therapy outcome. The sensitive and minute image patterns that cannot be discovered by the naked eye can predict therapy responders versus non-responders by utilizing AI-based algorithms. Accurate prediction of aggressive image features by non-invasive methods can be used to improve CAR-T cell therapeutic intervention. A flowchart depicting the use of AI for CAR-T cell therapy is shown in Fig. 3.

The challenge in building AI-based CAR-T cell prediction models is the requirement for large-omics and clinical datasets, necessitating the development of multi-omics datasets for implementing AI approaches. Radiomics and other cancer imaging data need to be generated to predict imaging signatures associated with CAR-T therapy response using various machine-learning techniques.

## Conclusions and future directions

**Fig. 4:**
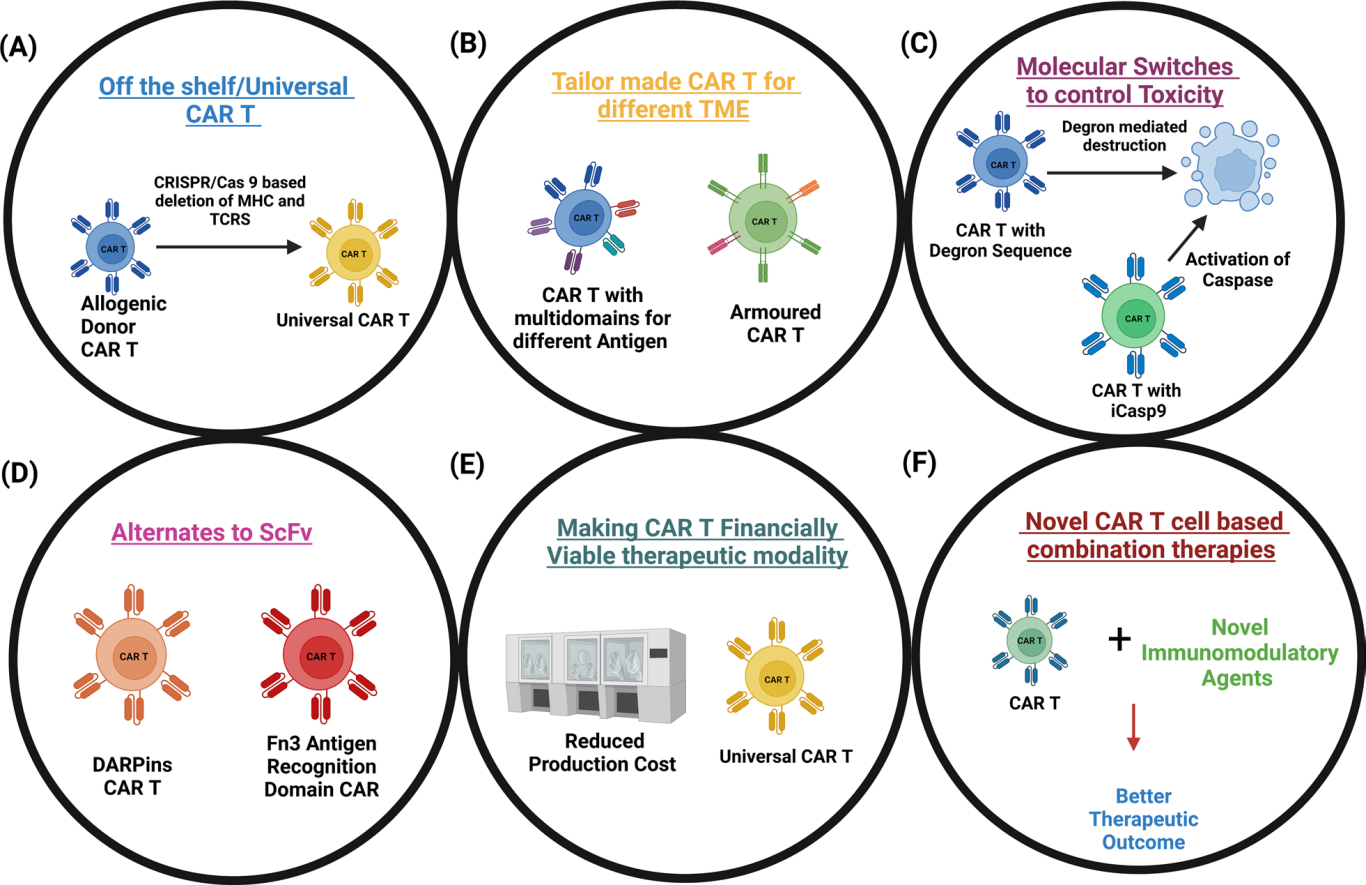
Building better CAR-T cells. **A** *Off-the-shelf/Universal CAR-T cells*, Generation of universal CAR-T cells from an allogenic donor by deletion of MHC and TCRs using CRISPR/Cas-9 technology. **B** *Tailor-made CAR-T cells for different TME.* Multidomain and Armored CAR-T cells to tackle different tumor microenvironments to stimulate CAR-T cell growth. **C** Molecular Switches to control CAR-T cell cytotoxicity, Programming of CAR-T cells with degron (degradation) sequence and iCasp9 (Inducible caspase 9) to induce their destruction upon life-threatening toxicity in treated patients. **D** *Alternate to ScFv.* Potential alternative to bulky ScFv like DARPin based CARs and Fn3 recognition domain CAR-T cells. **E** Making CAR-T cells financially viable therapeutic modality. Reduction cost of production and introduction of universal CAR-T cells, which can be produced in bulk. **F** Novel CAR-T cell based combination therapies. Combination CAR-T cell therapy with novel immunomodulatory agents will help in achieving a durable therapeutic response in cancer patients

CAR-T cell therapy has been successful in treating B-cell malignancies, particularly ALL. However, significant improvements are needed to enhance its efficacy in hematological malignancies and extend it to solid cancers. The US FDA has approved four CAR-T therapies targeting CD19 in B-ALL and B-NHL and two CAR-T cell therapies against BCMA in MM. The addition of costimulatory domains in second-generation CARs has improved the persistence of CAR-T cells in vivo, and newer generations are being developed to address the challenges posed by different types of TME (Fig. 4). Efforts are being made to develop multi-antigen-specific CAR-Ts to address the issue of tumor antigen escape. Humanizing scFv, which is a complicated process, can be replaced by the Type III domain of human fibronectin (Fn3) or the development of designed ankyrin repeat proteins (Fig. 4). Producing CAR-T cells is a complex and time-consuming procedure. However, the advent of CRISPR/Cas9 gene editing technology, enabling efficient knockout of cellular HLA and TCR, holds promise for revolutionizing the process. This approach is poised to significantly impact the cost-efficiency and methodology of CAR-T cell therapy development in the upcoming years (Fig. 4). While CAR-T cell therapies have not yet received approval for the treatment of solid tumors as of now, advancements in technology such as artificial intelligence and high-throughput screening, offer promising potential. These tools could expedite the discovery of unique and effective targets, not just for solid tumors but also for hematological malignancies. By integrating machine learning algorithms with extensive genomic and proteomic datasets, we could identify novel targets and predict patient responses to therapy, thereby expanding the applicability of CAR-T cell therapies in the future. The development of 4th and 5th generation CARs with encouraging results in solid cancer is an area that is being actively pursued [86, 254]. Incorporating molecular switches to fine-tune the activity of CAR-T cells and control their viability in circulation is also being investigated [62, 380]. The cost of CAR-T cell therapy remains a significant hurdle, but with its potential spreading to many centers around the globe, the cost is expected to decrease significantly. This expectation is based on the principle of economies of scale: as the production of CAR-T cell therapy increases, the cost per unit tends to decrease. This is due to the spreading of fixed costs over a greater volume and the improved efficiency and expertise gained from greater experience and specialization. Moreover, the cost of CAR-T cell therapy includes manufacturing costs, hospital admission costs, supportive care costs, and the cost of managing adverse events [381]. As more centers around the globe adopt this technology, increased competition and technological advancements are expected to drive down these costs.

Furthermore, as research continues to evolve, novel techniques that are more cost-effective may be developed, such as off-the-shelf CAR-T cells which are produced from healthy donors' T cells and could be used in multiple patients. This would greatly reduce the individual manufacturing costs associated with creating a unique CAR-T cell therapy for each patient [382]. Despite the challenges, CAR-T cell therapy is expected to revolutionize cancer treatment multiple ways such as targeted treatment, personalized treatment approach, durable response, potential in solid tumors, and as a living drug. However, challenges remain, including managing severe side effects, refining the treatment for use against a broader range of cancers, reducing the high costs associated with this personalized therapy, and finding ways to make the production process faster and more efficient.

Nevertheless, CAR-T cell therapy stands as a promising paradigm shift in cancer treatment, offering a potent, personalized, and potentially long-lasting method to combat this pervasive disease.

## Data Availability

Not applicable.
