# Correction: Harnessing the potential of CAR-T cell therapy: progress, challenges, and future directions in hematological and solid tumor treatments

**DOI:** 10.1186/s12967-023-04404-z

**Published:** 2023-08-25

**Authors:** Gunjan Dagar, Ashna Gupta, Tariq Masoodi, Sabah Nisar, Maysaloun Merhi, Sheema Hashem, Ravi Chauhan, Manisha Dagar, Sameer Mirza, Puneet Bagga, Rakesh Kumar, Ammira S. Al-Shabeeb Akil, Muzafar A. Macha, Mohammad Haris, Shahab Uddin, Mayank Singh, Ajaz A. Bhat

**Affiliations:** 1 Department of Medical Oncology (Lab.), Dr. BRAIRCH, All India Institute of Medical Sciences (AIIMS), New Delhi, Delhi, 110029 India; 2 Laboratory of Cancer Immunology and Genetics, Sidra Medicine, Doha, Qatar; 3 Department of Diagnostic Imaging, St. Jude Children’s Research Hospital, Memphis, TN USA; 4 National Center for Cancer Care and Research, Hamad Medical Corporation, 3050 Doha, Qatar; 5 Department of Human Genetics, Sidra Medicine, Doha, Qatar; 6 Shiley Eye Institute, University of California San Diego, San Diego, CA USA; 7 Department of Chemistry, College of Sciences, United Arab Emirates University, Al-Ain, United Arab Emirates; 8 School of Biotechnology, Shri Mata Vaishno Devi University, Katra, Jammu and Kashmir 182320 India; 9 Department of Human Genetics-Precision Medicine in Diabetes, Obesity and Cancer Program, Sidra Medicine, P.O. Box 26999, Doha, Qatar; 10 Watson-Crick Centre for Molecular Medicine, Islamic University of Science and Technology, Pulwama, Jammu and Kashmir India; 11 Center for Advanced Metabolic Imaging in Precision Medicine, Department of Radiology, Perelman School of Medicine, University of Pennsylvania, Philadelphia, USA; 12 Laboratory Animal Research Center, Qatar University, Doha, Qatar; 13 Translational Research Institute, Academic Health System, Hamad Medical Corporation, P.O. Box 3050, Doha, Qatar

**Correction: Journal of Translational Medicine (2023) 21:449** **https://doi.org/10.1186/s12967-023-04292-3**

Following publication of the original article [1], we have been notified that the author’s name was misspelled and affiliation 7 should be changed. Those should be as follows in this correction:

- First name: Maysaloun
- Last name: Merhi
- ^7^Department of Chemistry, College of Sciences, United Arab Emirates University, Al-Ain, United Arab Emirates
